# Impact of *Pediococcus acidilactici* GLP06 supplementation on gut microbes and metabolites in adult beagles: a comparative analysis

**DOI:** 10.3389/fmicb.2024.1369402

**Published:** 2024-04-03

**Authors:** Mengdi Zhao, Yuanyuan Zhang, Yueyao Li, Keyuan Liu, Kun Bao, Guangyu Li

**Affiliations:** ^1^College of Animal Science and Technology, Qingdao Agricultural University, Qingdao, China; ^2^College of Animal Science and Technology, Jilin Agriculture University, Changchun, China

**Keywords:** probiotic, gut microbiota, short-chain fatty acids, metabolome, beagle

## Abstract

There is growing interest in the potential health benefits of probiotics for both humans and animals. The study aimed to investigate the effects of feeding the canine-derived probiotic *Pediococcus acidilactici* GLP06 to adult beagles by analysing the microbiome and metabolome. Twenty-four healthy adult beagles were randomly assigned to four groups. The CK group received a standard diet, while the three probiotic groups, the LG group (2 × 10^8^ CFU/day/dog), MG group (2 × 10^9^ CFU/day/dog), and HG group (2 × 10^10^ CFU/day/dog), received the standard diet supplemented with varying amounts of probiotics. The results show that, compared to the CK group, total antioxidant capacity was significantly increased in the MG and HG groups (*p* < 0.05), and superoxide dismutase and catalase were significantly increased in the HG group (*p* < 0.05). Compared to the CK group, malondialdehyde and blood urea nitrogen content were significantly decreased in the MG and HG groups (*p* < 0.05). Additionally, secretory immunoglobulin A activity was significantly increased in the HG group compared to the CK and LG groups (*p* < 0.05), and immunoglobulin G activity was significantly increased in the HG group compared to the CK, LG, and MG groups (*p* < 0.05). In addition, compared with the CK group, the abundance of *Faecalitalea* and *Collinsella* increased in the LG group, and the relative abundance of *Tyzzerella* and *Parasutterella* increased in the MG group. The α diversity and the relative abundances of beneficial bacteria (*Faecalibacterium*, *Lachnospiraceae_NK4A1316*, and *Ruminococcaceae_UCG-005*) were higher in the HG group than in the CK group. Furthermore, acetic acid content was significantly increased in the HG group compared to the CK, LG, and MG groups (*p* < 0.05). Butyric acid, isobutyric acid, and the total SCFA content were significantly increased in the HG group compared to the CK group (*p* < 0.05). Moreover, metabolome analysis revealed 111 upregulated and 171 downregulated metabolites in the HG group. In conclusion, this study presents evidence that supplementing with *P. acidilactici* GLP06 can have a positive impact on antioxidant activity, immunoproteins, SCFAs, and gut microbiota in adult beagles. These findings highlight the potential of probiotics as a dietary intervention to enhance gut health and overall wellbeing in companion animals.

## 1 Introduction

In recent years, the pet food industry has experienced significant growth, driven by the increasing popularity of pet ownership and the rising demand for pet-related products and services (Samant et al., 2021). Pets are now regarded as beloved family members rather than just animals (Guo et al., 2022). Consequently, there has been a significant increase in the demand for pet products, such as natural pet food ingredients, functional pet food, and prescription pet food (Di Cerbo et al., 2017; Jian et al., 2022). Probiotics, as one of the principal products in functional foods, have been used in human and animal husbandry and have also attracted the focus of the pet industry (Grześkowiak et al., 2015).

“Live microorganisms that, when administered in adequate amounts, confer a health benefit on the host” is the definition of probiotics (Hill et al., 2014). Previous studies have shown that probiotics can modulate immune function, interact with the host gut microbiota, enhance the integrity of the intestinal barrier, and produce metabolites such as short-chain fatty acids (SCFAs), extracellular polysaccharides, and bacteriocins (van Baarlen et al., 2013; Aoudia et al., 2016; La Fata et al., 2018; Sanders et al., 2019). Lactic acid bacteria (LAB) have been reported to be among the safest probiotics, including *Lactobacillus*, *Pediococcus*, *Streptococcus*, *Bifidobacterium*, and *Streptocnccaceae* (Sanders et al., 2019). *Pediococcus acidilactici,* which belongs to the genus *Pediococcus*, has been reported to have probiotic potential, including the alleviation of anxiety, maintenance of intestinal homeostasis, protection of the intestinal tract, and antioxidant properties (Ruiz-Moyano et al., 2011; Liu et al., 2020; Bai et al., 2021; Tian et al., 2021).

Weaned piglets fed *P. acidilactici* FT28 had better apparent total tract digestibility (ATTD), blood biochemistry, and antioxidant status (Dowarah et al., 2018). *P. acidilactici* has also been reported to improve constipation in mice (Qiao et al., 2021). In addition, a study reported positive effects of canine-derived probiotics on faecal SCFAs and cell-mediated immune responses in healthy dogs (Kumar et al., 2017). On the other hand, host-derived microorganisms are preferred as probiotics compared to non-host-source microorganisms. This is because they are very familiar with the intestinal tract environment, are more adherent and persistent, and host-derived microorganisms have evolved to be more adapted to the gastrointestinal environment of the host gut (Lee et al., 2017; Dowarah et al., 2018; Johnson et al., 2023). Therefore, there is a need to explore the impact of host-derived probiotics on pet health.

However, the effectiveness of *P. acidilactici* GLP06, which was isolated from the gastrointestinal tract of healthy canines, on gastrointestinal health and metabolism has not been reported. We hypothesised that supplementing with canine-derived *P. acidilactici* GLP06 would improve faecal scores and ATTD and have a positive effect on the gastrointestinal environment of canines. The aim of this study was to assess the impact of supplementing probiotic GLP06 on ATTD, nitrogen (N) metabolism, serum antioxidants, immune protein activities, gut microbiota, SCFAs, and metabolism in adult beagles.

## 2 Materials and methods

The laboratory animals needed for this study were approved by the Laboratory Animal Ethics Committee of Qingdao Agricultural University (grant No. DWKJ202307043; Qingdao, China).

### 2.1 Experimental strain

*P. acidilactici* GLP06, used in this study, was isolated from the gastrointestinal tract of healthy beagles and completed the probiotic potential and safety evaluation in our laboratory (Zhao et al., 2023). It was kept in the China Centre for Type Culture Collection (CCTCC; Wuhan, China) under the accession number CCTCC No. M2023200.

In this experiment, *P. acidilactici* GLP06 was cultured in Man, Rogosa, and Sharpe (MRS) broth (Solarbio, China) and passaged three times. The cells were then inoculated into MRS broth at a concentration of 2.0% (v/v) for 20 h. Afterwards, they were centrifuged at 9391× *g* (10,000 rpm in Centrifuge 5,430 Eppendorf, Germany) for 10 min at 4°C. After removing the supernatant, the cells were resuspended in PBS (0.1 mol/L, pH = 7.2), and the bacterial concentration was adjusted to 1 × 10^10^ CFU/mL.

### 2.2 Experimental design and feeding management

The study included 24 adult beagles with an average age of 3.71 ± 1.09 years and a body weight of 17.24 ± 2.66 kg. The study enrolled participants with body condition scores (BCS) of 6.2 ± 0.85 (Laflamme, 1997). They were randomly assigned to four groups, with six individuals per group (three females and three males in each group). The no-probiotic-added group (CK) received a standard diet, whereas the three probiotic groups (low-dose GLP06 group [LG] with 2 × 10^8^ CFU/day/dog, medium-dose GLP06 group [MG] with 2 × 10^9^ CFU/day/dog, and high-dose GLP06 group [HG] with 2 × 10^10^ CFU/day/dog) received the standard diet supplemented with different amounts of probiotics. The study lasted for 5 weeks, with the first week being an adaptation period. During the second week, the beagles were gavaged with 2 mL of probiotics per day according to the experimental design, while the CK group received the same volume of PBS through gavage.

To achieve clinical effects, probiotic concentrations should be at least 1 × 10^6^ CFU/mL in the small bowel and 1 × 10^8^ CFU/g in the colon (Minelli and Benini, 2008). Kumar et al. (2017) administered a dose of canine-derived *Lactobacillus johnsonii* CPN23 at 2–3 × 10^8^ CFU/day/dog to adult dogs, which positively affected hindgut fermentation metabolites and cell-mediated immune responses. On the other hand, there are reports of clinical conditions where probiotics are effective in treating antibiotic-associated diarrhoea only at higher doses (>1 × 10 ^10^ CFU/day/dog; Ouwehand, 2017). Similarly, researchers supplemented adult beagles with *Weissella Cibaria* JW15 at levels of 1.5 × 10^10^ CFU/day/dog and 1.5 × 10^11^ CFU/day/dog, which improved lipid parameters and improved outcomes in adult dogs (Sun et al., 2019). Combining the references and the cost of probiotics (the higher the dosage administered, the higher the cost), this experiment was designed with a minimum dose of 2 × 10^8^ CFU/day/dog, and the maximum dose was set at 2 × 10^10^ CFU/day/dog.

Prior to the start of the trial, the beagles received vaccinations and were regularly dewormed. All kennels were located in the same environmentally controlled room (21.0 ± 1.0°C) with a 12-h light and a 12-h dark cycle. Each dog was housed in a separate cage. The cages were disinfected once a week with the compound hydrogen peroxide solution (Aladdin, China). Although animals were housed and fed individually, they were allowed to exercise and play outside of their cages (with people and toys) in the animal room for several hours, at least three times a week. Based on the maintenance energy requirements of adult dogs (National Research Council, 2006), data from previous feeding records, and an energy estimate from the diet, provide enough food to maintain body weight. Maintaining each animal’s body weight requires weekly, or even more frequent, adjustments to the amount of feed. Dogs had free access to fresh water at all times.

The respiration rate, temperature, and pulse were recorded weekly (Xu et al., 2019). Meanwhile, the faecal scores were recorded daily. The faeces of the beagles were evaluated for sensory characteristics using the Waltham^®^ faeces scoring system (WFS; Supplementary Figure S1; Fournier et al., 2021). The following scale was used during faeces consistency observations: 1 = crumbles with little pressure; 1.5 = hard and dry, stool cracks when pressed; 2.0 = well formed, does not leave a mark when picked up; 2.5 = well formed with a slightly moist surface, leaves a mark when picked up; 3.0 = moist, beginning to loose form, leaving a definite mark when picked up; 3.5 = very moist, still with some definite form; 4.0 = most or all form is lost, no real shape; 4.5 = liquid stool with slight consistency; 5.0 = entire liquid stool.

### 2.3 Ration composition and nutrient levels

The standard diet for the experiment was formulated according to the National Research Council (2006), and the composition and nutritional levels of the standard diets are presented in Table 1.

**Table 1:** Composition and nutrient levels of the diets (air-dry basis, %).

Ingredients	Content	Nutrient levels^b^	Content
Extrusion corn	24	Crude protein	27.22
Extruded soybean	8	Ether extract	9.72
Corn germ meal	30	Crude ash	8.63
Fish meal	5	Carbohydrate	54.43
Meat and bone meal	3	Crude fibre	3.30
Chicken meal	12	GE/(MJ/kg)	19.65
Duck meal	12	ME/(MJ/kg)	16.99
Spray-dried blood cells	0.8	DE/(MJ/kg)	16.71
Chicken oil	2	Ca	0.86
CaHPO_4_	0.8	TP	0.57
Lys	0.9	Lys	1.34
Met	0.5	Met	0.80
Premix^a^	1	Cys	0.18
Total	100.00	Arg	0.90

^a^One kilogram of premix contained the following: vitamin A 625,000 IU, vitamin D_3_ 100,000 IU, vitamin E 6,000 IU, vitamin K_3_ 200 mg, vitamin B_1_ 1,250 mg, vitamin B_2_ 900 mg, vitamin B_6_ 750 mg, vitamin B_12_ 2.25 mg, biotin 10 mg, folic acid 150 mg, nicotinic acid 2,500 mg, calcium pantothenate 1,750 mg, vitamin C 10,050 mg, choline 240,000 mg, Fe (FeSO_4_) 9,600 mg, Cu (CuSO_4_) 1,800 mg, Zn (ZnSO_4_) 7,800 mg, Mn (MnSO_4_) 4,800 mg, KI 144 mg, Se (Na_2_SeO_3_) 30 mg. ^b^GE, DE, and ME were calculated values, whereas the others were measured values. ^c^GE, gross energy; DE, digestive energy; ME, metabolic energy.

### 2.4 Sample collection

On the morning of day 28 of the experiment, fresh faeces (within 15 min) were collected in frozen tubes (Axygen, United States) and immediately stored at −80°C for microbiological analyses, SCFA content, and non-targeted metabolite assays. Additionally, 10 mL of venous blood was collected from the forelimbs of the beagles. Serum was collected by centrifugation at 4°C, 464× *g* (1,800 rpm, Centrifuge 5,702, Eppendorf, Germany) for 10 min (Kostanjšak et al., 2022; Zentrichová et al., 2023).

The total faeces were collected from the 25th to the 28th day of the experiment, weighed, and frozen at −20°C. Similarly, the total urine was collected from days 25 to 28 and recorded as the total volume. The urine samples were filtered through filter paper and stored at −20°C until the tests were analysed. After the experiment, all the faeces collected from each beagle were mixed thoroughly. Two hundred grams of faeces were weighed and dried at 65°C for 72 h until a constant weight was achieved. The faeces were then crushed (Retsch BB50, DEU), passed through a 40-mesh sieve, and stored for testing.

### 2.5 Indicators and methods of measurement

#### 2.5.1 Apparent total tract digestibility and nitrogen metabolism

Dry matter (DM; AOAC 934.01) and crude ash (ASH; AOAC 942.05) determinations of the samples were conducted following the AOAC method (Horwitz and Latimer, 2006). The N content was determined using the Automatic Kjeldahl Nitrogen Determination (FOSS 8400, DK; Etheridge et al., 1998). The crude protein (CP) content was obtained by calculating the N content multiplied by 6.25. The ether extract (EE) content was determined by the Soxhlet fat extraction method (Haineng SOX606, China; Nielsen, 2010). The calcium (Ca) content was assessed using the ethylenediaminetetraacetic acid disodium salt (EDTA) titration method (Belyea et al., 1976). The total phosphorus (TP) content was determined using the ammonium molybdate method (AOAC 995.11). The crude fat (*CF*; AOAC 962.09) contents were measured using an Automatic Fibre Tester (ANKOM A2000i, United States), while the amino acid contents were analysed using a fully automatic amino acid analyser (Hitachi L-8800, Japan).

Feed energy was calculated, and the formulas for the ATTD and nitrogen metabolism-related indices for each nutrient are shown in Supplementary Material 1.

#### 2.5.2 Serum biochemical indicators

The detection kit (Nanjing Jiancheng Bioengineering Institute, China) was used to measure the total antioxidant capacity (T-AOC), superoxide dismutase (SOD), glutathione peroxidase (GSH-Px), catalase (CAT), malondialdehyde (MDA), aspartate aminotransferase (AST), alanine aminotransferase (ALT), and blood urea nitrogen (BUN) contents in the serum of beagles. Simultaneously, enzyme-linked immunosorbent assay (ELISA) kits (Jiangsu Meimian, China) were selected to detect immunoglobulin G (IgG) and secretory immunoglobulin A (sIgA). Because *P. acidilactici* GLP06 was administered via gavage and expected to primarily act at the mucosal level, sIgA was analysed in faeces. The operating procedures strictly followed the kit instructions, and data were measured using an enzyme marker (Tecan, Switzerland).

#### 2.5.3 16S rRNA sequencing

Genomic DNA from faecal samples was extracted using the E.Z.N.A.^®^ Stool DNA Kit (Omega Bio-Tek, United States). Then, the V3–V4 region of the bacterial 16S rRNA gene was amplified using universal primers. The PCR products were then visualised on a 1.0% agarose gel (TSJ001, Tsingke, China), and the nucleic acids were purified using an Agencourt AMPure XP kit (Beckman Coulter, United States). A library was constructed using the NEBNext Ultra II DNA Library Prep Kit (New England Biolabs, United States). Finally, the library was sequenced using a NovaSeq 6,000 SP Reagent Kit v1.5 (Illumina, United States). The similarity threshold for OTU clustering was set at 97% (Stackebrandt and Goebel, 1994). One dog in the CK group and one in the HG group failed the faecal sample quality control, resulting in only five beagles in each of the CK and HG groups for which results were available.

#### 2.5.4 Short-chain fatty acids

Determination of fatty acids in beagle faeces by LC–MS/MS. Briefly, 30–40 mg of frozen faecal samples were placed into a 1.5-ml centrifuge tube. Then, 1 mL of 50% acetonitrile (ACN, Fisher Chemical, USA) was added, followed by 2–3 metal grinding beads. The sample was processed in an E6618 tissue grinder (Beyotime, China) for 1 min at 60 Hz and then centrifuged at 15,871× *g* (13,000 rpm in a Centrifuge 5,430 Eppendorf, Germany) for 10 min at 4°C. One hundred microliters of supernatant was taken and diluted proportionally to 10 mg of sample per 1.8 mL of 50% ACN solution. The mixture was vortexed and shaken (Scilogex, United States) for 30 s and then centrifuged at 15,871× *g* for 30 s. Twenty microliters of the supernatant was aspirated, and 10 μL of 200 mM 3-nitrophenylhydrazine-HCl (3-NPH-HCl, Sigma-Aldrich, United States) was added separately. Ten microlitres of 200 mM N-(3-dimethylaminopropyl)-N′-ethylcarbodiimide-HCl (EDC-HCl, Sigma-Aldrich, United States), 80 μL of 50% ACN, 50 μL of 7% pyridine (Sigma-Aldrich, United States), and 1 μL of isotope internal standard solution (Toronto Research Chemicals, Canada) were vortexed and shaken for 3 min, derivatised in a constant temperature water bath at 40°C for 30 min and centrifuged at 15,871× *g* for 1 min at 4°C. Finally, 20 μL of the reaction solution after derivatisation was added to 280 μL of 50% ACN, vortexed for 30 s, centrifuged at 15,871× *g* for 10 min at 4°C, aspirated into the injection vial, and then subjected to LC–MS/MS analysis (LC-30 HPLC, SCIEX QTRAP 5500 mass spectrometry, Phenomenex: Kinetex C18, 2.6 μm 100 × 3.00 mm, column temperature: 40°C, flow rate: 0.7 mL/min).

#### 2.5.5 Untargeted metabolomics

50 mg of faecal samples were loaded into 1.5 mL EP tubes with 600 μL of pre-cooled MeOH (Fisher Chemical, United States): ACN (Thermo Fisher Scientific, United States): H_2_O (Thermo Fisher Scientific, United States) solution containing internal standards (v:v:v = 2:2:1). Two steel beads were added, and the tissue grinder was ground for 120 s at 60 Hz. Ultrasonication (PS-60AL, Leidebang, China) was carried out for 10 min, and then the sample was kept at −20°C for 1 h. Centrifugation was performed for 15 min at 15,871× *g* and 4°C, and 200 μL of the sample was freeze-dried using a CentriVap (Labconco, United States). Next, 200 μL of ACN:H_2_O solution (v:v = 1:1) was added to resolubilise the mixture. It was then shaken for 30 s, sonicated for 10 min, and incubated at 20°C for 2 h before being centrifuged at 15,871× *g* at 4°C for 15 min. Finally, 150 μL of the supernatant was aspirated for liquid chromatography–tandem mass spectrometry (LC-30, Shimadzu, and TripleTOF 5,600+, SCIEX; Fu et al., 2023). The cardinal criteria used for screening differentially abundant metabolites were *p* < 0.05, VIP > =1, and fold change <0.67 or > 1.5. The screening criteria for the chord plot included a correlation coefficient |*r*| > 0.8 and *p* < 0.05.

### 2.6 Data analysis

Data were expressed as the mean ± standard error of the mean (SEM), visualised utilising GraphPad Prism (8.3.0), one-way analysis of variance (ANOVA) of SPSS (version 25.0), and Dunnett’s multiple comparison test for statistical analysis, with significant differences between groups (*p* < 0.05).

## 3 Results

### 3.1 Physiological indices and faecal scores of beagles

**Figure 1:**
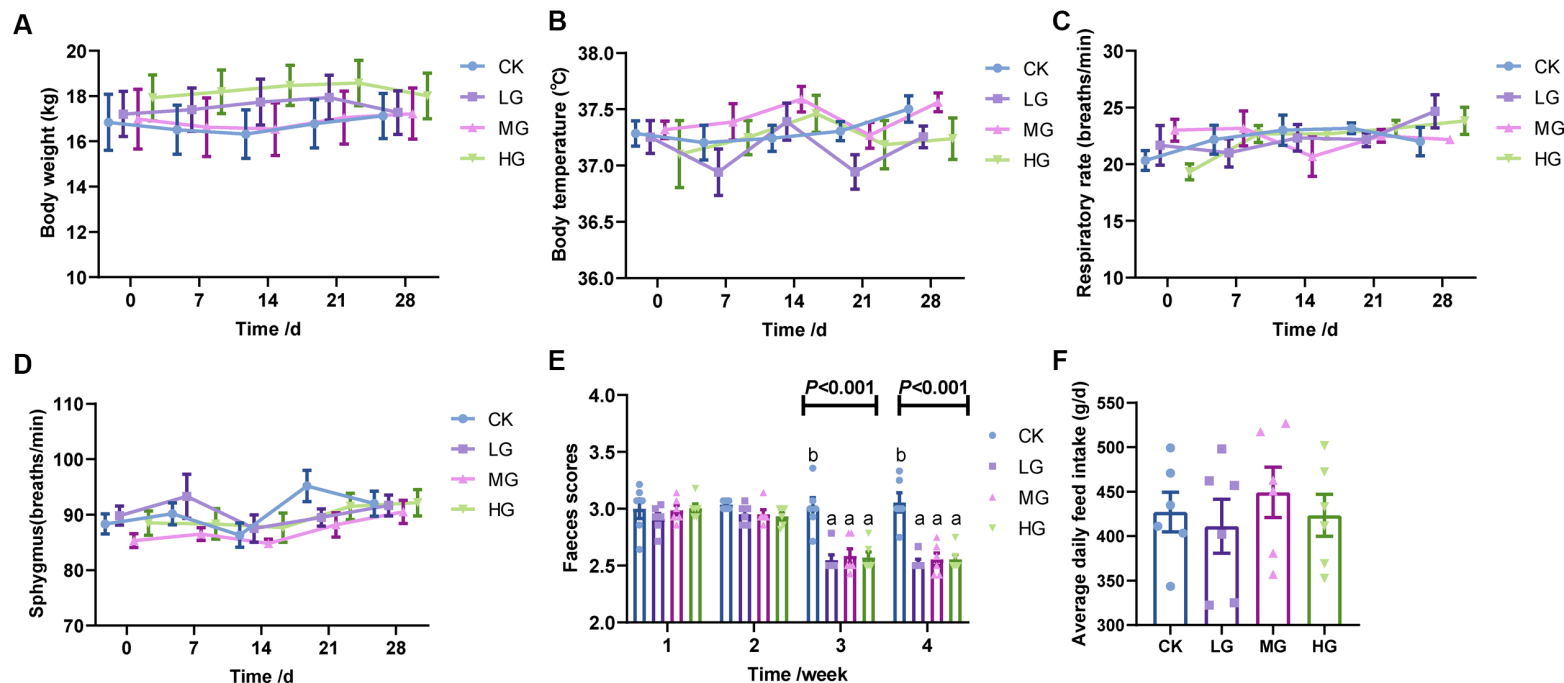
Health status, faecal scores, and average daily feed intake in probiotic-supplemented beagles. **(A)** body weight; **(B)** body temperature; **(C)** respiration; **(D)** sphygmus; **(E)** faecal scores; and **(F)** average daily feed intake in beagles. Values were displayed as the mean ± SEM, n = 6.

The body temperature, respiration rate, and pulse rate of beagles were all within the normal range of 36.9–37.6°C, 19.3–24.6 beats/min, and 84.8–95.2 breaths/min, respectively, and did not differ significantly between treatments (*p* > 0.05; Figures 1A–D).

Regarding faecal scores, there were no significant differences between the groups during the initial 2-week supplementation period (0–14 days). However, after the third and fourth weeks (15–28 days), the faecal scores of beagles in the probiotic-supplemented group significantly differed from those in the CK group (*p* < 0.05; Figure 1E). There were no significant differences found between the four groups in the average daily feed intake of the beagles (*p* > 0.05; Figure 1F).

### 3.2 Apparent total tract digestibility and nitrogen metabolism of beagles

Analysis of the ATTD of DM, CP, EE, ASH, or carbohydrate to probiotics in beagle dogs showed that the probiotic-fed group was similar to the CK group (*p* > 0.05; Table 2). Similarly, there were no significant differences in nitrogen intake, faecal nitrogen, urinary nitrogen, retained nitrogen, net protein utilisation (NPU), or biological value (BV) between the groups supplemented with probiotics and the CK group (*p* > 0.05; Supplementary Table S1).

**Table 2:** Effect of supplemented different concentrations of probiotic GLP06 on the ATTD of adult beagles (%).

Items	Dry matter	Crude protein	Ether extract	Crude ash	Carbohydrate
CK	78.46	79.34	94.98	36.09	81.79
LG	79.43	80.82	94.94	38.97	82.39
MG	77.51	77.92	93.61	32.48	81.56
HG	80.63	80.52	95.34	43.67	83.93
SEM	0.72	0.90	0.35	1.90	0.56
*p*-value	0.48	0.69	0.32	0.20	0.47

Values were displayed as the mean ± SEM, *n* = 6.

### 3.3 Serum antioxidants and immune proteins

**Figure 2:**
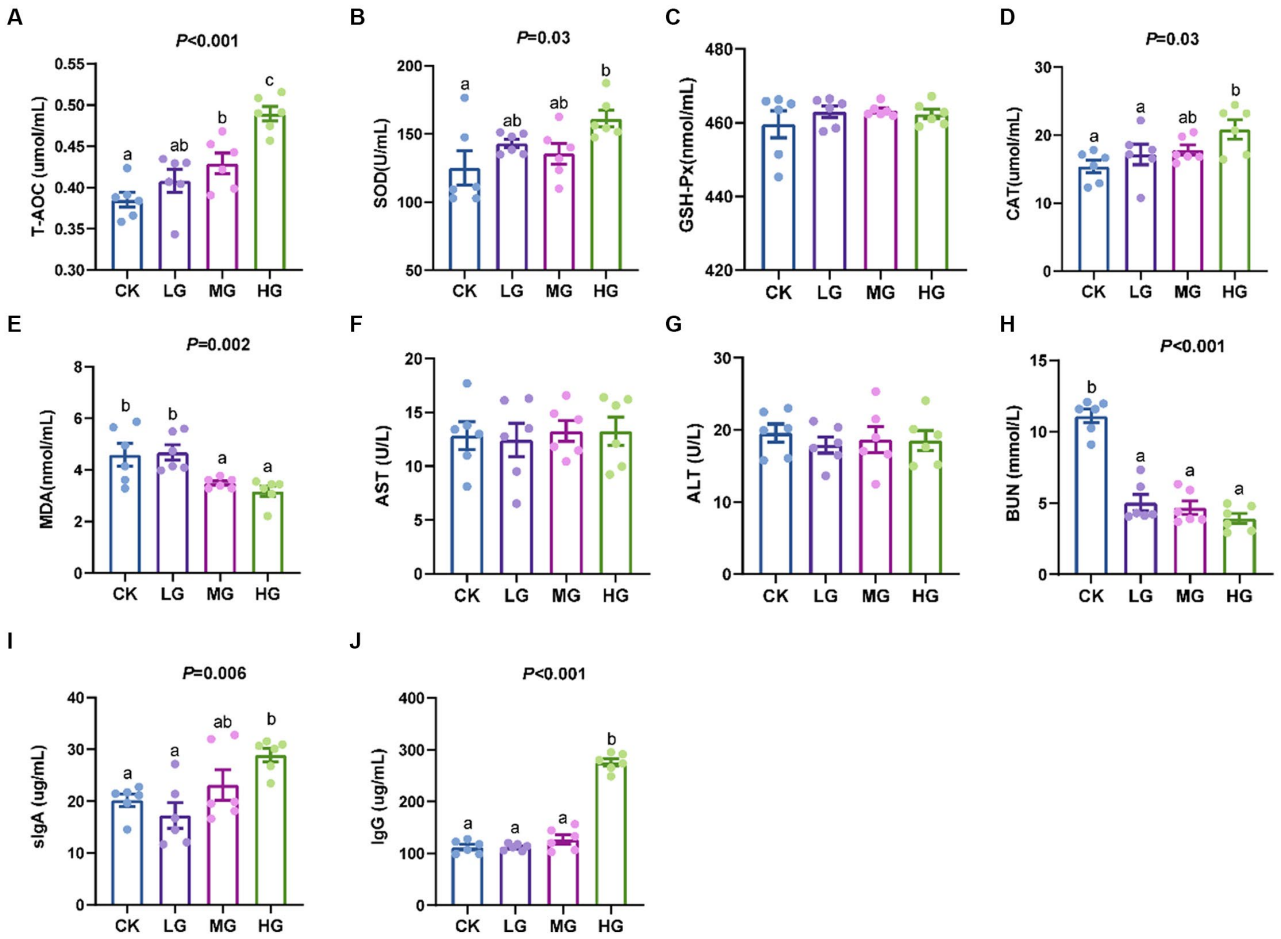
Serum indices in probiotic-fed beagles. **(A)** total antioxidant capacity; **(B)** superoxide dismutase; **(C)** glutathione peroxidase; **(D)** catalase; **(E)** malondialdehyde; **(F)** aspartate aminotransferase; **(G)** alanine aminotransferase; **(H)** blood urea nitrogen in beagles; **(I)** sIgA; and **(J)** IgG. Values were displayed as the mean ± SEM, *n* = 6.

Compared to the CK group, the HG group increased the activities of T-AOC, SOD, CAT, sIgA, and IgG (*p* < 0.05; Figures 2A,B,D,I,J) and decreased the levels of MDA and BUN (*p* < 0.05; Figures 2E,H). However, probiotic treatment did not affect the activities of GSH-Px, AST, and ALT (*p* > 0.05; Figures 2C,F,G).

### 3.4 Short-chain fatty acids

To analyse the effect of *P. acidilactici* GLP06 on the secretion of SCFAs, this study measured the content of SCFAs in faeces. Notably, the acetic acid and butyric acid contents of beagles in the HG group were significantly higher than those in the CK and LG groups (*p* < 0.05; Table 3). Furthermore, the total SCFA content in the HG group was significantly higher compared to the CK group (*p* < 0.05), but there was no significant difference compared to the LG and MG groups (*p* > 0.05). In addition, compared to the CK group, the HG group showed a significant increase in isobutyric acid (*p* < 0.05). There were no significant differences in the contents of propionic acid, 2-methylbutyric acid, isovaleric acid, and total branched-chain fatty acids (BCFAs) of the beagles in four groups (*p* > 0.05).

**Table 3:** Effect of supplemented different concentrations of probiotic GLP06 on short-chain fatty acids in adult beagles (mg/g DM faeces).

Items	Acetic acid	Propionic acid	Butyric acid	Total SCFA^1^	Isobutyric acid	2-Methylbutyric acid	Isovaleric acid	Total BCFA^2^
CK	6.06^a^	4.01	0.84^a^	10.90^a^	0.40^a^	0.29	0.48	1.17
LG	7.09^a^	4.99	0.91^a^	12.99^ab^	0.43^a^	0.29	0.58	1.31
MG	7.41^a^	4.80	1.13^ab^	13.35^ab^	0.53^ab^	0.39	0.72	1.64
HG	9.23^b^	5.23	1.49^b^	15.96^b^	0.70^b^	0.48	0.75	1.93
SEM	0.37	0.20	0.09	0.62	0.04	0.03	0.05	0.12
*P*-value	0.012	0.149	0.048	0.026	0.048	0.093	0.212	0.109

^a^Total SCFA content = acetate acid + propionate acid + butyrate acid content. ^b^Total BCFA content = isobutyric acid + 2-methylbutyric acid + isovaleric acid content. Values were displayed as the mean ± SEM, *n* = 6.

### 3.5 Gut microbial diversity and composition

**Figure 3:**
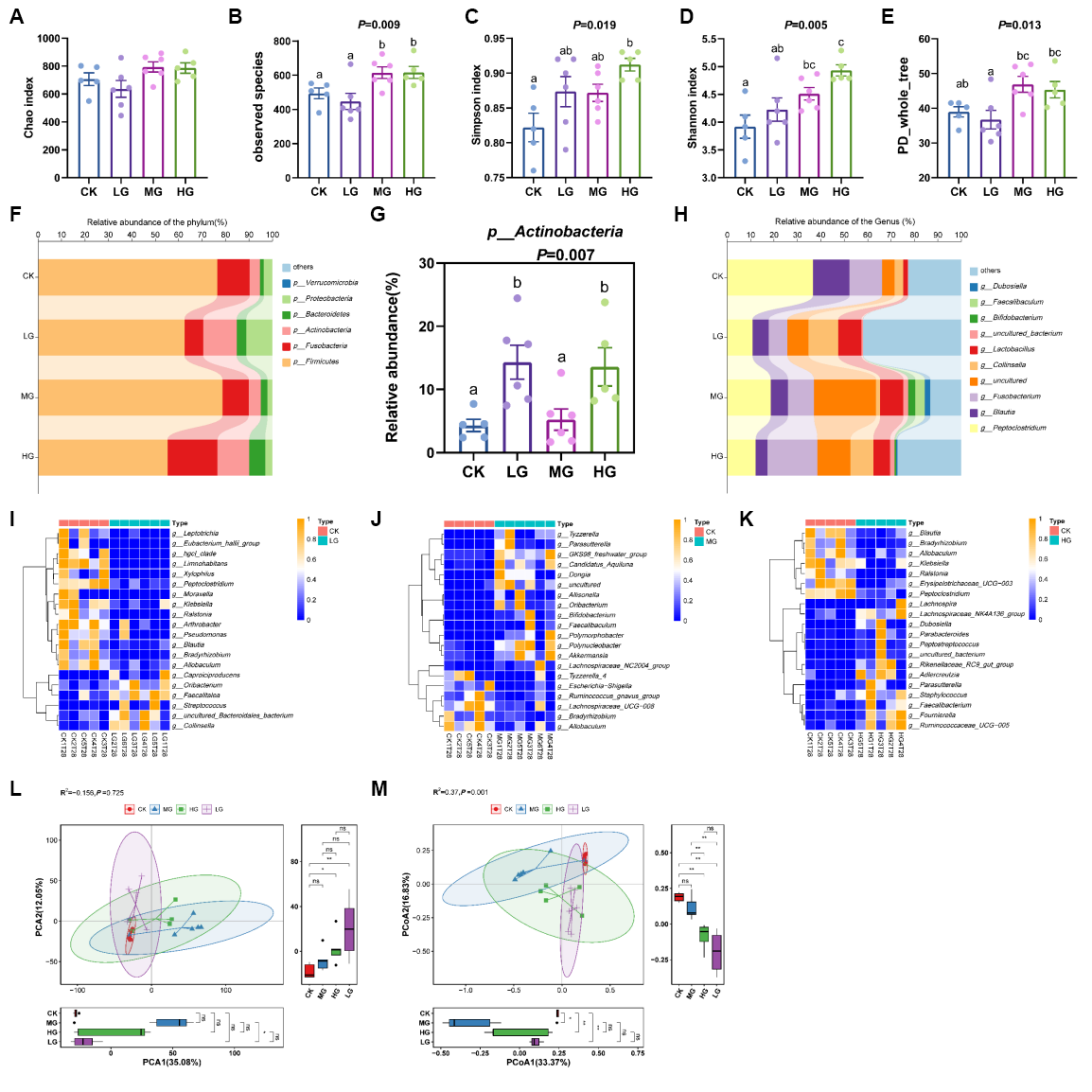
Gut microbiota of beagles supplemented with different concentrations of the probiotic GLP06. **(A)** Chao index; **(B)** observed species; **(C)** Simpson; **(D)** Shannon; **(E)** PD-whole-tree; **(F)** relative abundance of the phylum; **(G)** relative abundance of *p_Actinobacteria*; **(H)** relative abundance of the genus; **(I)** genus abundance between CK and LG groups; **(J)** genus abundance between CK and MG groups; **(K)** genus abundance between CK and HG groups using the Wilcoxon test; **(L)** principal component analysis; and **(M)** PCOA. Values were displayed as the mean ± SEM, *n* = 5 or *n* = 6.

The sequencing coverage of the groups of samples was good, the amount of sequencing data was large enough, the species were more dispersed, and the sampling was more adequate (Supplementary Figure S2). Analysis of α-diversity showed that the observed species and Shannon indices were considerably higher in the MG and HG groups than in the CK group (*p* < 0.05; Figures 3B,D). The Simpson index was significantly higher in the HG group than in the CK group (*p* < 0.05), but the Simpson index was not significantly different in the MG and LG groups than in the CK group (*p* > 0.05; Figure 3C). However, the Chao index and PD-whole-tree in the LG, MG, and HG groups were similar to those of the CK group (*p* > 0.05; Figures 3A,E). Although there was no significant difference, there seemed to be a trend of improvement in the HG group. In addition, the relative abundance of the four groups at the phylum level is shown in Figure 3F, and the abundance of *Actinobacteria* was markedly increased in the LG and HG groups compared to the CK group (*p* < 0.05; Figure 3G). At the genus level, as shown in Figure 3H, the abundance of *Faecalitalea* and *Collinsella* increased in the LG group compared with the CK group (Figure 3I). The relative abundances of *Tyzzerella* and *Parasutterella* increased in the MG group (Figure 3J). Additionally, *Faecalibacterium*-, *Lachnospiraceae_NK4A136*-, and *Ruminococcaceae_UCG-005*-relative abundances increased in the HG group (Figure 3K; Supplementary Figure S3). Additionally, the principal component analysis showed that PCA1 (35.08%) component MG group differed significantly from the LG group (*p* < 0.05; Figure 3L) and PCA2 (12.05%) component MG and HG groups differed significantly from the CK group (*p* < 0.05). Principal co-ordinates analysis showed that PCoA1 (33.37%) component LG, MG, and HG groups differed significantly compared to the CK group (*p* < 0.05; Figure 3M), and PCoA2 (16.83%) component HG and LG groups of the CK group differed significantly (*p* < 0.05).

### 3.6 Prediction of metabolomic function

**Figure 4:**
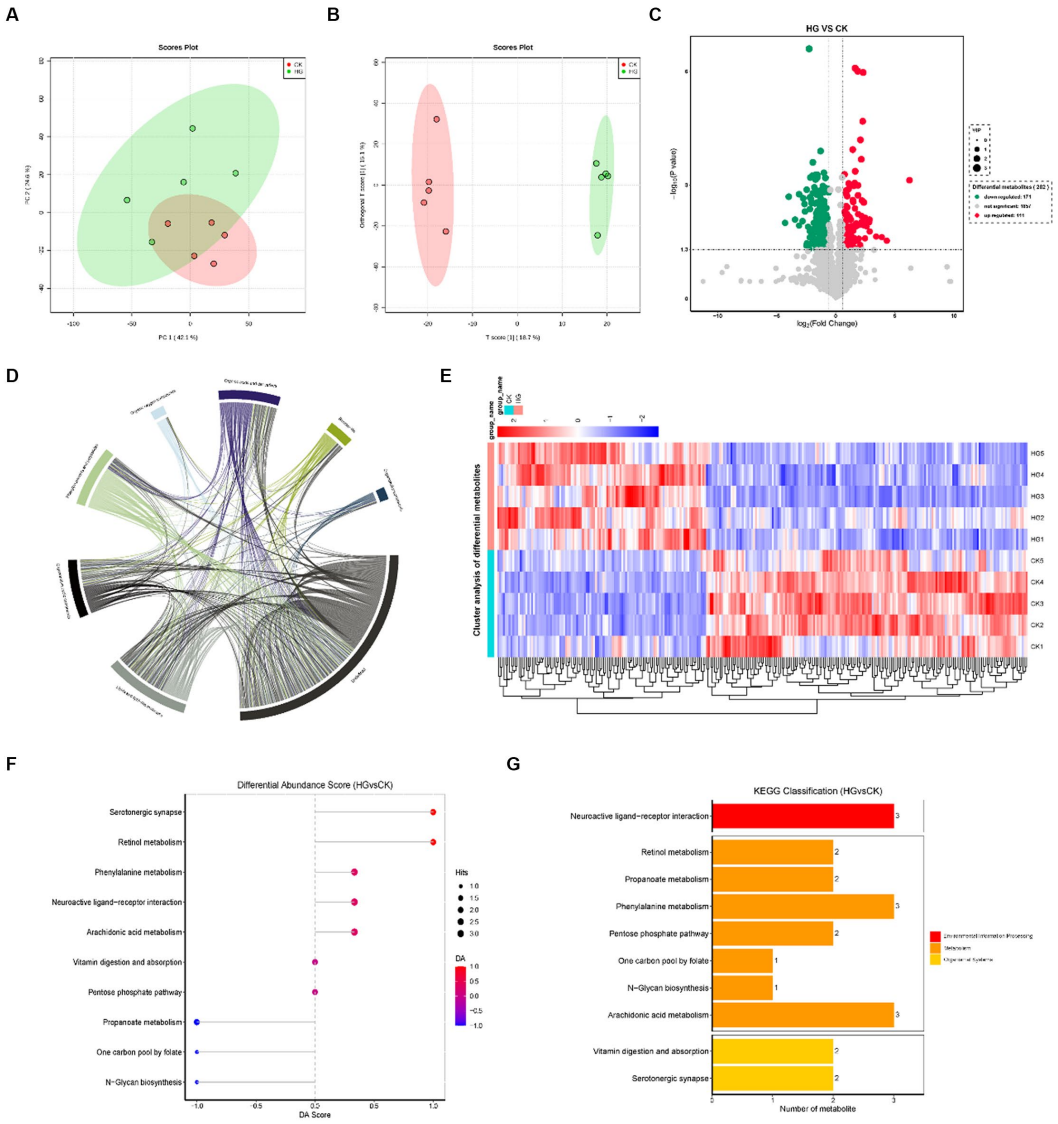
Faeces untargeted metabolome in beagles supplemented with different concentrations of probiotic GLP06. **(A)** principal component analysis; **(B)** orthogonal partial least squares discrimination analysis; **(C)** volcano plot; **(D)** chord diagram; **(E)** clustering heatmap; **(F)** differential abundance score of KEGG metabolic pathways; and **(G)** KEGG metabolic pathway classification histogram, *n* = 5.

The between-group differences in the PCA score plots show a less pronounced separation (Figure 4A). However, the OPLS-DA model revealed more significant differences between groups (Figure 4B). In this study, a total of 2,139 metabolites were detected in the HG vs. CK group. Of these, 111 metabolites were upregulated differentially and 171 were downregulated (Figures 4C,E; Supplementary Table S2). The chord diagram illustrates that the metabolites are mainly associated with lipids, lipid-like molecules, organic acids and derivatives, organ heterocyclic compounds, phenylpropanoids and polyketides, and organic oxygen compounds (Figure 4D). Metabolites with similar characteristics were grouped together, and the variation in metabolites between the HG and CK groups is shown in Figure 4E. KEGG pathway analyses revealed that probiotic GLP06 mainly influences the serotonergic synapse (prostaglandin B_2_, PGB_2_, and prostaglandin D_2_, PGD_2_), retinol metabolism (retinyl ester, RE, and all-*trans*-4-oxoretinoic acid), and phenylalanine metabolism (3-phenylpropionic acid) pathways (Figures 4F,G).

### 3.7 Correlation between metabolites and microbial genus

**Figure 5:**
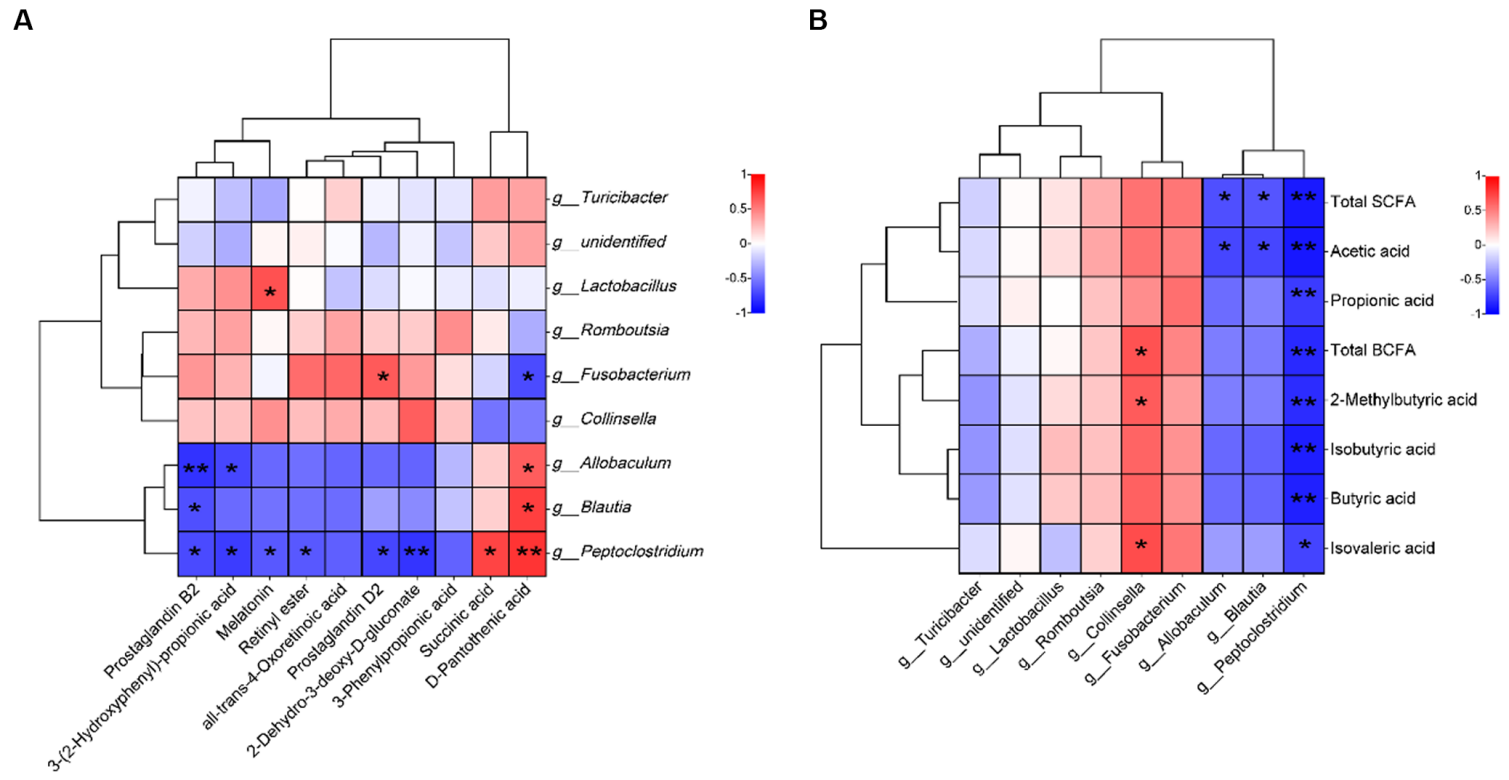
Spearman correlation analysis. **(A)** correlation between metabolic differentiators and microbial genus level and **(B)** correlations between short-chain fatty acids and microbial genus level. Red and blue grids indicate positive and negative correlations, respectively (*p < 0.05 and **p < 0.01), *n* = 5.

The correlation between metabolic differentiators and the microbial genus level showed that PGD_2_ was positively correlated with *Fusobacterium* (Figure 5A, *p* < 0.05) and had a negative correlation with *Peptoclostridium* (*p* < 0.05). Additionally, retinyl ester showed negative correlations with *Peptoclostridium* (*p* < 0.05), and PGB_2_ displayed negative correlations with *Allobaculum*, *Blautia*, and *Peptoclostridium* (*p* < 0.05 and *p* < 0.01).

The correlation between short-chain fatty acids and the microbial genus level is depicted in Figure 5B. *Collinsella* was positively correlated with BCFAs, 2-methylbutyric acid, and isovaleric acid (*p* < 0.05). *Allobaculum* and *Blautia* were negatively correlated with total SCFAs and acetic acid (*p* < 0.05). Conversely, *Peptoclostridium* was negatively correlated with all SCFAs (*p* < 0.05 and *p* < 0.01).

## 4 Discussion

Previous studies have demonstrated that *P. acidilactici* can improve host gut microbiota, stimulate various non-specific immunities, and inhibit the growth of pathogenic bacteria in the gut intestinal tract (Ferguson et al., 2010; Fernandez et al., 2016; Kim et al., 2018; Zhang et al., 2022). Regarding *P. acidilactici* GLP06, our previous study has shown that canine-derived *P. acidilactici* GLP06 has probiotic potential and antioxidant capacity and is safe and free of drug-resistance genes (Zhao et al., 2023). In this study, neither ATTD nor nitrogen metabolism was significantly altered in beagles fed with *P. acidilactici* GLP06. A previous study found that feeding probiotics did not alter the ATTD of beagles (de Lima et al., 2020). However, previous studies have reported that feeding probiotics GBI-30 (10^9^ CFU/mL) had a positive effect on the ATTD of beagles compared to the CK group. However, this effect was eliminated by decreasing the concentration of the probiotic (Panasevich et al., 2021). This discrepancy could be attributed to differences in probiotic strains and concentrations, diet composition, and nutritional levels, but further trials are needed to verify this. In addition, in the present study, faecal scores were reduced (less moisture, harder) in the probiotic-fed group after 14 days but were within the desirable range (2.5–3.0 on a 5-point scale) both before and after feeding (Lee et al., 2022).

SOD, CAT, and GPX are the first line of antioxidant defence of the organism and play an indispensable role in the overall antioxidant defence (Ighodaro and Akinloye, 2018). Meanwhile, MDA is a marker for lipid peroxidation and oxidative stress (Del Rio et al., 2005). *P. acidilactici* has been reported to have the ability to increase the antioxidant resistance of the host organism (Hoseinifar et al., 2017). In this study, beagles fed *P. acidilactici* had higher serum levels of T-AOC, SOD, and CAT but lower levels of MDA, confirming that feeding *P. acidilactici* GLP06 improved the resistance to oxidative stress in beagles. The significance of this finding is that it may be essential to alleviate the stress response in pets during transport or environmental changes. Environmental stressors can stimulate cells to produce reactive oxygen species, which disrupt the antioxidant defence system and induce the onset of an inflammatory response (Medzhitov, 2008; Herzog et al., 2014). Liver function (AST and ALT) and kidney function (BUN) indices of beagles were also examined in this study, and the results demonstrated that *P. acidilactici* GLP06 had no adverse effects on liver and kidney function. Another important finding is that oral administration of GLP06 significantly reduced serum levels of BUN in mice, similar to a previous report. Researchers fed *P. acidilactici* NJB421 to mice with ochratoxin A-induced intoxication and found that it alleviated ochratoxin A-induced oxidative stress and liver injury and significantly reduced BUN levels (Tang et al., 2023).

Probiotics modulate the innate and adaptive immune systems of the host, which is crucial for stimulating the production of intestinal antibodies, especially IgA (Yan and Polk, 2011). Symbiotic bacteria provide intestinal immunity by regulating IgA secretion, and IgA deficiency appears to be associated with chronic enteropathy in dogs (Littler et al., 2006; Fagarasan, 2008). IgG is a necessary glycoprotein for protecting against invading pathogens and has anti-inflammatory and immunomodulatory functions (Lux et al., 2010). In this study, feeding *P. acidilactici* GLP06 increased the concentrations of serum IgG and faecal sIgA in beagles. According to these data, we can infer that *P. acidilactici* GLP06 perhaps promotes the immune system’s ability to better fine-tune the microbial balance in the gut of beagles (Rollenske et al., 2021). Previous studies have demonstrated that supplementation with *P. acidilactici* ZPA017 increased IgA and IgG concentrations in weaned piglets (Liu et al., 2020). In addition, feeding a blend of probiotics significantly increased serum IgG and faecal sIgA levels in elderly canines, promoting a shift towards a younger gut microbiota (Xu et al., 2019).

SCFAs are crucial for gut integrity and can regulate metabolic health by modulating gastrointestinal pH, fuelling epithelial cells, and participating in different host signalling mechanisms (Blaak et al., 2020). The fermentation of dietary fibre by the intestinal phylum (*Firmicutes* and *Bacteroidetes*) produces SCFAs, with acetic, propionic, and butyric acids accounting for over 95% of the total, along with BCFAs (isobutyric, 2-methylbutyric, and isovaleric acids, among others), which, although present in low abundance, also have biological effects (Krautkramer et al., 2021). In this study, acetic acid, butyric acid, and isobutyric acid levels were significantly higher in the oral high-dose probiotic group. Butyric acid is a preferred energy source for colonic epithelial cells. It also helps maintain intestinal barrier function and regulates immunity, oxidative stress, and anti-inflammation (Bedford and Gong, 2018; Fu et al., 2019). The metabolites of *P. acidilactici* GLP06 are predominantly acetic acid with only small amounts of butyric acid, and the significant increase in butyric acid may be the enhancement of butyrate production in the gut through cross-feeding of acetic acid with another commensal microbiota (den Besten et al., 2013). However, there is a minimal metabolic exchange between propionate and acetate, which may explain why no significant changes in propionate were observed in this study.

In this study, the primary phyla of the canine gut microbiota were *Firmicutes*, *Fusobacteria*, *Actinobacteria*, *Bacteroidetes*, and *Proteobacteria*, which is consistent with previous reports (Hayasaka et al., 2021). Additionally, the α-diversity (observed species, Simpson and Shannon indices) of the gut microbiota of beagles was significantly higher in the HG group than in the CK group. The observed species and Shannon indices were increased in the MG group. These results indicate that feeding GLP06 is not only safe for beagles but also improves the homeostasis of the gastrointestinal environment. It is reported that dogs with enteritis develop ecological dysregulation, characterised by decreased bacterial diversity and abundance (Minamoto et al., 2015, 2019). Moreover, compared to the CK group, the abundance of *Faecalitalea* and *Collinsella* increased in the LG group, and the relative abundance of *Tyzzerella* and *Parasutterella* increased in the MG group. *Faecalitalea* and *Collinsella* have been reported as producers of butyrate (Zhou et al., 2021). In this study, an increase in butyric acid content was also observed in the LG group of beagles compared to the CK group. It is reported that *Parasutterella* are all asaccharolytic and producers of succinate (Ju et al., 2019). Succinic acid is one of the key metabolites produced by gut microbes and plays an important role in the cross-feeding of SCFA (Fischbach and Sonnenburg, 2011). This may explain the different levels of acetic acid and butyric acid content in the MG group compared to the control group. Furthermore, the relative abundance of *Actinobacteria, Faecalibacterium*, *Lachnospiraceae_NK4A1316*, and *Ruminococcaceae_UCG-005* was significantly higher in the HG group than in the CK group. *Actinobacteria* can produce SCFAs and play a beneficial role in maintaining the intestinal barrier (Binda et al., 2018). *Lachnospiraceae_NK4A1316* is a member of the *Lachnospiraceae* family, *Firmicutes* phylum. This family ferments dietary polysaccharides to produce SCFAs and is negatively correlated with various metabolic and chronic diseases (Truax et al., 2018; Hu et al., 2019). Moreover, *Ruminococcaceae_UCG-005* can maintain intestinal health by metabolising butyrate and other SCFAs (Gu et al., 2022). The bacteria have the metabolic capability to produce SCFAs, which coincides with the increased SCFA content in the HG group of beagles. These results suggest that *P. acidilactici* GLP06 can enhance beagle SCFA content and improve gastrointestinal health by regulating gut microbes.

In this study, significant upregulation of the serotonergic synapse, retinol metabolism, and phenylalanine metabolism pathways was observed in the HG group. Prostaglandins are a class of lipids produced through the enzymatic metabolism of arachidonic acid, including PGB_2_ and PGD_2_. A previous study reported that PGD_2_ increased hydrogen peroxide production and antioxidant enzyme expression in mice (Loupp et al., 2015). Notably, among the altered metabolites, retinyl ester is one of the most abundant forms of retinol in the body, including palmitic, oleic, stearic, and linoleic acid (LA; O’Byrne and Blaner, 2013). It is important to note that LA is known as an essential fatty acid in dogs and plays a vital role in the skin barrier and the prevention of skin diseases (Watson et al., 2018). In addition, retinol is enzymatically activated into retinoic acid (RA) through a two-step oxidation process. It is well known that RA is an active metabolite of vitamin A and essential for immune cell development, differentiation, apoptosis, and function (Larange and Cheroutre, 2016; Erkelens and Mebius, 2017). Another important finding is that 3-phenylpropionic acid was upregulated in the phenylalanine metabolism pathway. A recent study found that *B. fragilis*-derived 3-phenylpropionic acid enhanced the host intestinal epithelial barrier by activating intestinal epithelial AhR signalling (Hu et al., 2023). In this study, the probiotic GLP06 further enhanced the protective effect of the intestinal barrier on the host by targeting and modulating the gut microbiota and increasing the levels of metabolites.

One limitation of the study that should be noted is that we only studied the gastrointestinal environment of healthy beagles, and our findings cannot yet be generalised to puppies or older dogs with gastrointestinal problems or those who are prone to diarrhoea.

## 5 Conclusion

Despite these limitations, our study demonstrates that supplementing with *P. acidilactici* GLP06 can have a positive impact on faecal scores, serum antioxidant activity, immunoproteins, SCFAs, and gut microbiota in adult beagles, leading to improved gut homeostasis. These findings underscore the potential of *P. acidilactici* GLP06 as a dietary intervention to enhance gut health in companion animals. In the future, further research is needed to clarify the underlying mechanisms and optimise probiotic formulations for specific gastrointestinal conditions in more vulnerable populations, such as puppies or senior dogs.

## Data availability statement

The datasets presented in this study can be found in online repositories. The names of the repository/repositories and accession number(s) can be found in the article/Supplementary material.

## Ethics statement

The animal study was approved by the Laboratory Animal Ethics Committee of Qingdao Agricultural University. The study was conducted in accordance with the local legislation and institutional requirements.

## Author contributions

MZ: Data curation, Formal analysis, Investigation, Methodology, Software, Visualization, Writing – original draft, Writing – review & editing. YZ: Data curation, Software, Visualization, Writing – original draft. YL: Data curation, Software, Visualization, Writing – original draft. KL: Formal analysis, Methodology, Writing – review & editing. KB: Formal analysis, Methodology, Writing – review & editing. GL: Conceptualization, Funding acquisition, Project administration, Resources, Writing – original draft, Writing – review & editing.
