# Supplementary material for: Impact of Pediococcus acidilactici GLP06 supplementation on gut microbes and metabolites in adult beagles: a comparative analysis
Source: Front Microbiol. 2024 Apr 3;15:1369402. doi: 10.3389/fmicb.2024.1369402 (PMC11021720; doi:10.3389/fmicb.2024.1369402)
Supplement: Supplementary file 1 [file Data_Sheet_1.docx]

**Supplementary material 1**

The apparent total digestibility and nitrogen metabolism-related indices for each nutrient were calculated as follows:

Gross energy (GE) (MJ/kg) = (5.7×crude protein + 9.4×ether extract + 4.1×carbohydrate) × 4.184

Digestive energy (DE) (MJ/kg) = GE × [(91.2-1.43 × crude fibre)/100]

Metabolic energy (ME) (MJ/kg) = DE - 1.04 x crude protein

Carbohydrate (%) = dry matter % - crude protein % - ether extract % - Ash %

ATTD of dry matter (%) = [(dry matter intake - dry matter in faeces)/dry matter intake] x 100

ATTD of certain nutrients (%) = [(intake of certain nutrients - amount of certain nutrients in faeces)/intake of certain nutrients] × 100

Net protein utilisation (%) = (Nitrogen deposition/food intake of nitrogen) × 100

Biological value of protein (%) = [nitrogen deposition/ (ingested nitrogen - faeces nitrogen)] × 100.

**Supplementary material 2**

**STable 1.** Effect of supplemented different concentrations of probiotic GLP06 on nitrogen metabolism in adult beagles

| Items | Intake [nitrogen](javascript:;)/(g/d) | Feces [nitrogen](javascript:;)/(g/d) | Urinary [nitrogen](javascript:;)/(g/d) | Retention [nitrogen](javascript:;)/(g/d) | NPU/% | BV of protein/% |
| --- | --- | --- | --- | --- | --- | --- |
| CK | 9.59 | 1.95 | 1.20 | 6.43 | 66.14 | 83.29 |
| LG | 8.14 | 1.58 | 1.43 | 5.12 | 62.53 | 77.16 |
| MG | 9.63 | 2.14 | 1.17 | 6.31 | 64.98 | 83.60 |
| HG | 7.91 | 1.54 | 1.73 | 4.63 | 58.36 | 72.13 |
| SEM | 0.36 | 0.11 | 0.13 | 0.35 | 1.94 | 2.20 |
| *P*-value | 0.19 | 0.15 | 0.42 | 0.18 | 0.53 | 0.20 |

NPU: Net protein utilisation; BV: Biological value of protein. Values were displayed as the mean ± SEM, n=6.

**STable 2**. Summary of differential metabolites.

| **Name** | **RT [min]** | **Formula** | **Molecular Weight** | **HMDB** | **PubChem** | **KEGG** |
| --- | --- | --- | --- | --- | --- | --- |
| Erucicoyl-EA | 8.5644 | - | - | - | - | - |
| Anhydroamarouciaxanthin B | 7.9586 | C40H50O3 | 578.3759 | 0036911 | 131752087 | NA |
| FAHFA(16:0/9-O-18:0) | 7.5110 | C34H66O4 | 538.4961 | 0112106 | 72189985 | NA |
| "7alpha,12alpha-Dihydroxy-5beta-cholestan-3-one" | 7.2837 | C27H46O3 | 418.3446 | 0006887 | NA | C05453 |
| Dolichyl beta-D-glucosyl phosphate | 6.9938 | C21H39O9P | 466.2331 | 0001054 | 22833557 | C01246 |
| Garcinol | 6.9565 | C38H50O6 | 602.3607 | 0252641 | 3531 | C09929 |
| LysoPC(O-18:0) | 6.9075 | C26H56NO6P | 509.3845 | 0011149 | 2733532 | C04317 |
| Ganoderiol I | 6.8575 | C31H50O5 | 502.3658 | 0037778 | 15602270 | NA |
| Dronedarone | 6.8467 | C31H44N2O5S | 556.2970 | 0251621 | 208898 | NA |
| Oleanolic acid | 6.8398 | C30H48O3 | 456.3603 | 0002364 | 10494 | C17148 |
| Ganoderiol G | 6.8388 | C31H52O5 | 504.3814 | 0037780 | 15602268 | NA |
| GlcADG 46:3 | 6.8375 | - | - | - | - | - |
| PA 42:3 | 6.8047 | - | - | - | - | - |
| MGDG 38:5 | 6.7919 | - | - | - | - | - |
| 10-Apo-beta-caroten-10-al | 6.7339 | C27H36O | 376.2766 | 0059605 | 6450190 | NA |
| LPI 18:1 | 6.7075 | - | - | - | - | - |
| Retinyl ester | 6.6527 | C20H30O2 | 302.2245 | 0003598 | 5460164 | C02075 |
| PA 27:2 | 6.6358 | - | - | - | - | - |
| GlcADG 32:3 | 6.4776 | - | - | - | - | - |
| 13-Carboxy-gamma-tocotrienol | 6.3807 | C28H40O4 | 440.2926 | 0012558 | 53481464 | NA |
| LPE 16:0 | 6.1633 | - | - | - | - | - |
| PA 27:0 | 6.0781 | - | - | - | - | - |
| "3alpha,7alpha,24(S)-trihydroxy-5beta-cholestan-27-al" | 5.8836 | C27H46O4 | 434.3396 | 0062208 | 56927975 | NA |
| cis-5-Tetradecenoylcarnitine | 5.8212 | C21H39NO4 | 369.2879 | 0002014 | 22833575 | NA |
| Diosgenin 3-O-beta-D-glucopyranoside | 5.7738 | C33H52O8 | 576.3662 | 0029310 | 12314556 | C20709 |
| Cohumulone | 5.7612 | C20H28O5 | 348.1936 | 0033981 | NA | NA |
| Neomycin B | 5.6968 | C23H46N6O13 | 614.3122 | 0015129 | 8378 | C01737 |
| PE 18:2e | 5.5427 | - | - | - | - | - |
| "3b,7b-Dihydroxy-5-androsten-17-one" | 5.4645 | C19H28O3 | 304.2038 | 0004624 | 9817954 | NA |
| Myristoylglycine | 5.2356 | C16H31NO3 | 285.2303 | 0013250 | 72348 | NA |
| 6beta-Hydroxyasiatic acid | 5.1406 | C30H48O6 | 504.3450 | 0036670 | 258809 | NA |
| "2-(Methoxycarbonyl)-5-methyl-2,4-bis(3-methyl-2-butenyl)-6-(2-methyl-1-oxopropyl)-5-(4-methyl-3-pentenyl)cyclohexanone" | 5.0977 | C29H46O4 | 458.3396 | 0035995 | 73106016 | NA |
| "15-Deoxy-Delta12,14-PGJ2" | 5.0613 | C20H28O3 | 316.2038 | 0005079 | 5311211 | C14717 |
| 12(R)-Hydroxyeicosatetraenoic Acid [12(R)-Hete] | 4.9890 | - | - | - | - | - |
| 1-Palmitoylglycerophosphoinositol | 4.8861 | C25H49O12P | 572.2961 | 0061695 | NA | NA |
| FAHFA 29:2 | 4.8706 | - | - | - | - | - |
| 2-tetracosanamidoethanesulfonic acid | 4.7923 | - | - | - | - | - |
| Cucurbitacin D | 4.7620 | C30H44O7 | 516.3087 | 0034695 | 5353546 | C08796 |
| LPG 24:4 | 4.7591 | - | - | - | - | - |
| all-trans-4-Oxoretinoic acid | 4.7513 | C27H46O3 | 418.3446 | 0006280 | 6437063 | C16678 |
| Gingerdione | 4.7473 | C17H24O4 | 292.1674 | 0039275 | 162952 | C10459 |
| "(3beta,5alpha,9alpha,22E,24R)-3,5,9-Trihydroxy-23-methylergosta-7,22-dien-6-one" | 4.7040 | C29H46O4 | 458.3396 | 0032669 | 131751277 | NA |
| FAHFA 29:6 | 4.7039 | - | - | - | - | - |
| Prostaglandin B2 | 4.6857 | C20H30O4 | 334.2144 | 0004236 | 5288144 | C05954 |
| Norethisterone | 4.6741 | C20H26O2 | 298.1932 | 0014855 | 6230 | C05028 |
| PC 22:5e | 4.5978 | - | - | - | - | - |
| PA 44:6 | 4.5765 | - | - | - | - | - |
| Diisooctyl phthalate | 4.5718 | C24H38O4 | 390.2770 | 0251353 | 33934 | C14577 |
| "PC(14:1(9Z)/22:2(13Z,16Z))" | 4.5716 | C44H82NO8P | 783.5778 | 0007921 | 52922294 | NA |
| Colneleate | 4.5329 | C18H30O3 | 294.2194 | 0030995 | 6441681 | C19827 |
| "4alpha-Methyl-5alpha-ergosta-8,14,24(28)-trien-3beta-ol" | 4.5160 | C29H46O | 410.3548 | 0304204 | 4489455 | C11508 |
| Rhipocephalin | 4.4174 | C21H28O6 | NA | NA | 11902 | C09714 |
| "(3beta,17alpha,23R)-17,23-Epoxy-3,29-dihydroxy-27-norlanost-8-ene-15,24-dione" | 4.4151 | C29H44O5 | 472.3188 | 0035112 | 23132225 | NA |
| LPS 14:0 | 4.4101 | - | - | - | - | - |
| Cholest-4-en-3-one | 4.3723 | C27H44O | 384.3392 | 0000921 | 91477 | C00599 |
| Prostaglandin D2 | 4.3246 | C20H32O5 | 352.2249 | 0001403 | 448457 | C00696 |
| Carboxin | 4.3167 | C12H13NO2S | 235.0666 | 0249648 | 21307 | C11255 |
| 20-ethyl-PGE2 | 4.2660 | - | - | - | - | - |
| Cavipetin C | 4.2648 | C24H36O4 | 388.2613 | 0030366 | 14527070 | NA |
| Porrigenin A | 4.2625 | C27H44O5 | 448.3188 | 0032783 | 12312669 | NA |
| "N-(2-morpholin-4-ylethyl)-3-(2,3,5,9-tetramethyl-7-oxofuro[3,2-g] chromen-6-yl)propanamide" | 4.2576 | - | - | - | - | - |
| PC 20:2e | 4.2541 | - | - | - | - | - |
| Meteneprost | 4.2475 | C23H38O4 | 378.2770 | 0254509 | 3028542 | NA |
| Bisphenol A diglycidyl ether | 4.2471 | C21H24O4 | 340.1674 | 0032737 | 2286 | C14348 |
| Soyasaponin I | 4.2464 | C48H78O18 | 942.5188 | 0034649 | 122097 | C08983 |
| PA 44:7 | 4.2317 | - | - | - | - | - |
| "(1S,7S,8xi,9beta,17xi)-1-(beta-D-Allopyranosyloxy)-22-hydroxy-7-methoxy-9,10,14-trimethyl-4,9-cyclo-9,10-secocholesta-5,24-dien-23-yl beta-D-allopyranoside" | 4.2193 | - | - | - | - | - |
| PE 37:1 | 4.2191 | - | - | - | - | - |
| 12-Ketodeoxycholic acid | 4.2046 | C24H38O4 | 390.2770 | 0000328 | 3080612 | NA |
| Lyciumoside II | 4.1994 | C38H64O17 | 792.4143 | 0039576 | 131752684 | NA |
| PA 42:12 | 4.1002 | - | - | - | - | - |
| Melatonin | 4.0729 | C13H16N2O2 | 232.1211 | 0001389 | 896 | C01598 |
| "(6b,7b,13R)-6,7-Diacetoxy-8,14-labdadiene-13-ol" | 4.0479 | C24H38O5 | 406.2719 | 0035288 | 18008143 | NA |
| 3-GluA Soyasapogenol B | 4.0460 | - | - | - | - | - |
| Testosterone cypionate | 4.0444 | C27H40O3 | 412.2977 | 0251641 | 6012 | C08156 |
| lysoDGTS 22:7 | 4.0130 | - | - | - | - | - |
| "(2S,3S,9R,13R,14S,16R)-2,3,16-trihydroxy-17-[(2R)-2-hydroxy-6-methyl-3-oxohept-6-en-2-yl]-4,4,9,13,14-pentamethyl-1,2,3,7,8,10,12,15,16,17-decahydrocyclopenta[a]phenanthren-11-one" | 4.0050 | - | - | - | - | - |
| DGDG 39:2 | 3.9715 | - | - | - | - | - |
| 3-(Galactosyl(1-2) glucuronyl)-22-DDMP Soyasapogenol B | 3.9602 | - | - | - | - | - |
| Alliofuroside A | 3.9598 | C44H72O18 | 888.4718 | 0041051 | 4979367 | NA |
| PE 22:0e | 3.9549 | - | - | - | - | - |
| L-Urobilinogen | 3.9498 | C33H48N4O6 | 596.3573 | 0004157 | 9548718 | C05789 |
| PG 25:3 | 3.9436 | - | - | - | - | - |
| PG 25:4 | 3.9434 | - | - | - | - | - |
| L-Urobilin | 3.9430 | C33H42N4O6 | 590.3104 | 0004159 | 440785 | C05793 |
| MGDG 23:4 | 3.9385 | - | - | - | - | - |
| Adynerin | 3.9376 | C30H44O7 | NA | NA | 11038 | C08845 |
| (S)-Zearalanone | 3.8432 | C18H24O5 | NA | NA | 17395752 | C14754 |
| PA 42:11 | 3.7936 | - | - | - | - | - |
| "(17alpha,23S)-Epoxy-28,29-dihydroxy-27-norlanost-8-ene-3,24-dione" | 3.7834 | C29H44O5 | 472.3188 | 0035624 | 131751817 | NA |
| Yamogenin | 3.7813 | C27H42O3 | NA | NA | 11110 | C08918 |
| "3b-Hydroxy-6b-(3-chloro-2-hydroxy-2-methylbutanoyloxy)-7(11)-eremophilen-12,8b-olide" | 3.7668 | C20H29ClO6 | 400.1652 | 0041278 | 85161954 | NA |
| Cerberin | 3.7605 | C32H48O9 | NA | NA | 135626450 | C19984 |
| "(3beta,5xi,9xi)-3-{[2-O-(beta-D-Glucopyranosyl)-beta-D-glucopyranosyl]oxy}-23-hydroxyolean-12-en-28-oic acid" | 3.7479 | - | - | - | - | - |
| Soyasaponin III | 3.7468 | C42H68O14 | 796.4609 | 0034651 | 13326389 | C19865 |
| "19-hydroxy-18-[3,4,5-trihydroxy-6-(hydroxymethyl)oxan-2-yl]oxyicosanoic acid" | 3.7278 | - | - | - | - | - |
| C75 | 3.7273 | - | - | - | - | - |
| Anastrozole | 3.7224 | C17H19N5 | 293.1640 | 0015348 | 2187 | C08159 |
| Latanoprost (free acid)-d4 | 3.6701 | - | - | - | - | - |
| "(R)-3,4-Dihydro-2-methyl-2-(4,8,12-trimethyl-3,7,11-tridecatrienyl)-2H-1-benzopyran-6-ol" | 3.6589 | C26H38O2 | 382.2871 | 0036368 | 9929901 | NA |
| 17beta-Hydroxy-5alpha-androstan-3-one cyclohexanecarboxylate | 3.6549 | C26H40O3 | NA | NA | 17396280 | C15283 |
| "[(2S,3R,4S,5R,6R)-6-[[(2R,3R,4R,5S,6S)-3,5-dihydroxy-6-methyl-4-[(2S,3R,4S,5S,6R)-3,4,5-trihydroxy-6-(hydroxymethyl)oxan-2-yl]oxyoxan-2-yl]oxymethyl]-2-[2-(3,4-dihydroxyphenyl)-5,7-dihydroxy-4-oxochromen-3-yl]oxy-5-hydroxy-4-[(2S,3R,4S,5S)-3,4,5-trihydroxyoxan-2-yl]oxyoxan-3-yl] (E)-3-(4-hydroxyphenyl)prop-2-enoate" | 3.5752 | - | - | - | - | - |
| "1,11,21-trihydroxy-1,6,11,16,21,26-hexazacyclohentriacontane-2,5,12,15,22,25-hexone" | 3.5687 | - | - | - | - | - |
| (+/-)-Equol | 3.5295 | - | - | - | - | - |
| Adouetine X | 3.5236 | C28H44N4O4 | 500.3362 | 0034216 | 5316533 | C09993 |
| "PA(22:2(13Z,16Z)/0:0)" | 3.5192 | - | - | - | - | - |
| (-)-Dioxibrassinin | 3.5117 | C11H12N2O2S2 | 268.0340 | 0038634 | 11807698 | NA |
| Pyrrhoxanthinol | 3.5054 | C37H46O5 | 570.3345 | 0035696 | 131751849 | NA |
| Apigenin | 3.5030 | C15H10O5 | 270.0528 | 0002124 | 5280443 | C01477 |
| 4-Methylumbelliferyl acetate | 3.5022 | C12H10O4 | 218.0579 | 0032989 | 366 | C03837 |
| 3-Methylbenzyl alcolhol | 3.5022 | C8H10O | NA | NA | 9425 | C07216 |
| 3-Phenylpropionic acid | 3.5001 | C9H10O2 | 150.0680 | 0000764 | 107 | C05629 |
| beta-Zearalenol | 3.5000 | C18H24O5 | 320.1623 | 0041838 | 13932155 | C14751 |
| Steviobioside | 3.4937 | C32H50O13 | 642.3251 | 0036707 | 16401639 | NA |
| Paroxetine | 3.4902 | C19H20FNO3 | 329.1427 | 0014853 | 43815 | C07415 |
| PE 21:3 | 3.4477 | - | - | - | - | - |
| "(8aR,12S,12aR)-12-Hydroxy-4-methyl-4,5,6,7,8,8a,12,12a-octahydro-2H-3-benzoxecine-2,9(1H)-dione" | 3.4356 | - | - | - | - | - |
| Dynorphin A (6-8) | 3.4290 | C18H37N9O4 | 443.2968 | 0012932 | 53481551 | NA |
| 4-hydroxy Nonenal Glutathione | 3.3789 | C19H33N3O8S | 463.1988 | 0240578 | 92970719 | NA |
| Valganciclovir | 3.3772 | C14H22N6O5 | 354.1651 | 0015548 | 64147 | NA |
| Clovoxamine | 3.3554 | C14H21ClN2O2 | 284.1291 | 0250372 | 68717 | NA |
| Morphine | 3.3538 | C17H19NO3 | 285.1364 | 0014440 | 5288826 | C01516 |
| Propachlor ESA | 3.3329 | - | - | - | - | - |
| "3-(4-Hydroxy-3-methoxyphenyl)-1,2-propanediol 2-O-(galloyl-glucoside)" | 3.3277 | C23H28O13 | 512.1529 | 0036376 | 85236310 | NA |
| Pimethixene | 3.3182 | C19H19NS | 293.1238 | 0240247 | 4822 | NA |
| Coniferyl alcohol | 3.3100 | C10H12O3 | 180.0786 | 0012915 | 1549095 | C00590 |
| "1alpha-hydroxy-24-(dimethylphosphoryl)-25,26,27-trinorvitamin D3 / 1alpha-hydroxy-24-(dimethylphosphoryl)-25,26,27-trinorcholecalciferol" | 3.3050 | - | - | - | - | - |
| Dienestrol | 3.2822 | C18H18O2 | 266.1306 | 0015027 | NA | C08090 |
| Kanamycin A | 3.2559 | C18H36N4O11 | 484.2380 | 0015303 | 6032 | C01822 |
| "4,8 Dimethylnonanoyl carnitine" | 3.2243 | C18H35NO4 | 329.2566 | 0006202 | 53477801 | NA |
| "1alpha-hydroxy-18-[m-(1-hydroxy-1-ethylpropyl)-benzyloxy]-23,24,25,26,27-pentanorvitamin D3 / 1alpha-hydroxy-18-[m-(1-hydroxy-1-ethylpropyl)-benzyloxy]-23,24,25,26,27-pentanorcholecalciferol" | 3.1894 | - | - | - | - | - |
| CAY10580 | 3.1891 | - | - | - | - | - |
| Staurosporine | 3.1883 | C28H26N4O3 | NA | NA | 5165 | C02079 |
| Gentamicin C1a | 3.1587 | C17H20FN3O3 | 333.1488 | 0014630 | 51081 | NA |
| CAY10641 | 3.1577 | - | - | - | - | - |
| Austalide C | 3.1227 | C30H38O11 | 574.2414 | 0034075 | 131751523 | NA |
| Mandelonitrile | 3.1217 | C8H7NO | 133.0527 | 0060486 | 9548674 | C00561 |
| Lippioside I | 3.0918 | C25H30O13 | 538.1686 | 0034265 | 131751541 | NA |
| 4-Ethylphenol | 3.0802 | C8H10O | 122.0731 | 0029306 | 31242 | C13637 |
| 2-(4-Hydroxyphenl)-Ethonol | 3.0787 | C8H10O2 | 138.0680 | 0004284 | 10393 | C06044 |
| 3-(2-Hydroxyphenyl)-propionic acid | 3.0725 | C9H10O3 | 166.0629 | 0033752 | 873 | C01198 |
| "(5xi,9xi)-3-[(2-O-beta-D-glucopyranosyl-alpha-L-arabinopyranosyl)oxy]-23-hydroxy-Olean-12-en-28-oic acid" | 3.0467 | - | - | - | - | - |
| "PE(18:4(6Z,9Z,12Z,15Z)/22:6(4Z,7Z,10Z,13Z,16Z,19Z))" | 3.0433 | C45H70NO8P | 783.4839 | 0009210 | 52924469 | NA |
| 6-Hydroxyhexanoic acid | 3.0178 | C6H12O3 | 132.0786 | 0012843 | 14490 | C06103 |
| Lipoyllysine | 3.0101 | C14H26N2O3S2 | 334.1384 | 0012996 | 53481572 | NA |
| Entacapone | 2.9815 | C14H15N3O5 | 305.1011 | 0012226 | 5281081 | C07943 |
| Leurosine | 2.9584 | C46H56N4O9 | NA | NA | 11410 | C09219 |
| PS 22:2 | 2.9556 | - | - | - | - | - |
| "2-Phenylethyl 6-O-[(2S,3R,4R)-3,4-dihydroxy-4-(hydroxymethyl)tetrahydro-2-furanyl]-beta-D-glucopyranoside" | 2.9085 | - | - | - | - | - |
| "26,26,26,27,27,27-hexafluoro-1alpha,24-dihydroxyvitamin D3 / 26,26,26,27,27,27-hexafluoro-1alpha,24-dihydroxycholecalciferol" | 2.9051 | - | - | - | - | - |
| Epoxyfumitremorgin C | 2.9049 | C22H23N3O4 | 393.1688 | 0038643 | 131752409 | NA |
| PS 26:6 | 2.8891 | - | - | - | - | - |
| 8-Benzylcanadine | 2.8888 | - | - | - | - | - |
| Alangicine | 2.8725 | C28H36N2O5 | NA | NA | 11518 | C09327 |
| Sofalcone | 2.8719 | C27H30O6 | 450.2042 | 0042013 | 5282219 | NA |
| PS 32:10 | 2.7884 | - | - | - | - | - |
| Tragopogonsaponin J | 2.7619 | C57H84O22 | 1120.545 | 0037924 | 14827928 | NA |
| Indinavir-N-glucuronide | 2.7370 | - | - | - | - | - |
| Neriifolin | 2.7362 | C30H46O8 | NA | NA | 11069 | C08876 |
| KT 5720 | 2.7169 | - | - | - | - | - |
| PS 23:4 | 2.7077 | - | - | - | - | - |
| Jubanine B | 2.6985 | C43H47N5O6 | 729.3526 | 0030206 | 101316795 | NA |
| Methyl benzoate | 2.6960 | C8H8O2 | 136.0524 | 0033968 | 7150 | C20645 |
| Glycochenodeoxycholate 7-sulfate | 2.6890 | C26H43NO8S | 529.2709 | 0002496 | 259 | C15559 |
| Valorphin | 2.6886 | C44H60N8O12 | 892.4330 | 0059789 | 90470048 | NA |
| 2-Hydroxy-3-methylbutyric acid | 2.6536 | C5H10O3 | 118.0629 | 0000407 | 99823 | NA |
| Capecitabine | 2.6066 | C15H22FN3O6 | 359.1492 | 0015233 | 60953 | C12650 |
| Novobiocin sodium | 2.6032 | C31H36N2O11 | 612.2319 | 0015185 | 54675769 | C12609 |
| "9-Hydroxy-4-(3,7-dimethyl-2,6-octadienyloxy)-psoralen" | 2.6027 | C22H24O5 | 368.1623 | 0039057 | 6440422 | NA |
| "Galb1-3[Neu5Aca2,6]GalNAca-Thr" | 2.5928 | - | - | - | - | - |
| L-gamma-Glutamyl-S-allylthio-L-cysteine | 2.5264 | C11H18N2O5S2 | 322.0657 | 0038515 | 131752387 | NA |
| Pefloxacin | 2.5152 | C17H20FN3O3 | 333.1488 | 0014630 | 51081 | NA |
| Morellin | 2.4971 | C33H36O7 | 544.2461 | 0030794 | 6164465 | C10085 |
| Gibberellin A1 glucosyl ester | 2.4966 | C25H34O11 | 510.2101 | 0038609 | NA | NA |
| PS 20:3 | 2.4960 | - | - | - | - | - |
| "1alpha,25-dihydroxy-26,26,26,27,27,27-hexafluoro-16,17,23,23,24,24-hexadehydro-19-norvitamin D3 / 1alpha,25-dihydroxy-26,26,26,27,27,27-hexafluoro-16,17,23,23,24,24-hexadehydro-19-norcholecalciferol" | 2.4767 | - | - | - | - | - |
| Spironolactone | 2.4755 | C24H32O4S | 416.2021 | 0014565 | 5833 | C07310 |
| D-Pantothenic acid | 2.4283 | C9H17NO5 | 219.1106 | 0000210 | 6613 | C00864 |
| "3,4-Dihydroxy-tamoxifen" | 2.4212 | C26H29NO3 | 403.2147 | 0061087 | 10386463 | NA |
| PG 18:3 | 2.4106 | - | - | - | - | - |
| "26,26,26,27,27,27-hexafluoro-1alpha,25-dihydroxy-23,23,24,24-tetradehydrovitamin D3 / 26,26,26,27,27,27-hexafluoro-1alpha,25-dihydroxy-23,23,24,24-tetradehydrocholecalciferol" | 2.4092 | - | - | - | - | - |
| Asn Ile | 2.4081 | - | - | - | - | - |
| Cyclo[glycylprolyltryptophylglycyl-beta-hydroxy-3-(3-methyl-2-buten-1-yl)tyrosylglycylvalyl] | 2.4035 | - | - | - | - | - |
| Taurolithocholic acid 3-sulfate | 2.3998 | C26H45NO8S2 | 563.2586 | 0002580 | 440071 | C03642 |
| Ala-trp | 2.3910 | C14H17N3O3 | 275.1269 | 0013209 | 85362 | NA |
| CAY10633 | 2.3869 | - | - | - | - | - |
| Trilobolide | 2.3792 | C27H38O10 | NA | NA | 11754 | C09563 |
| Homotrypanothione | 2.3630 | C28H51N9O10S2 | NA | NA | 51090896 | C16567 |
| DGDG 20:5 | 2.3305 | - | - | - | - | - |
| TMC 126 | 2.3257 | C28H38N2O8S | NA | NA | 17396648 | C15659 |
| Dexamethasone | 2.3198 | C22H29FO5 | 392.1999 | 0015364 | 5743 | C15643 |
| Isomorellic acid | 2.3107 | C33H36O8 | 560.2410 | 0030734 | 12294 | NA |
| Telbivudine | 2.2740 | C10H14N2O5 | 242.0902 | 0015394 | 159269 | NA |
| "(1S,4aS,7S,7aS)-1-[(6-O-beta-D-glucopyranosyl-beta-D-glucopyranosyl)oxy]-4a,7a-dihydro-4-[(1R)-1-[[(2E)-3-(4-hydroxyphenyl)-1-oxo-2-propen-1-yl]oxy]ethyl]-5-oxo-Spiro[cyclopenta[c]pyran-7(1H),2(5H)-furan]-4-carboxylic acid" | 2.2581 | - | - | - | - | - |
| IRIFLOPHENONE TRIMETHYL ETHER | 2.2449 | - | - | - | - | - |
| 2-Dehydro-3-deoxy-D-gluconate | 2.2112 | C6H10O6 | NA | NA | 3504 | C00204 |
| Virginiamycin S1 | 2.1901 | C43H49N7O10 | 823.3540 | 0030520 | 122731 | C11269 |
| Glucuronosyletoposide | 2.1600 | C35H39O19 | NA | NA | 13423 | C11245 |
| "(2S,9R,10R)-2,7,9,10-tetrahydro-10-hydroxy-9-methoxy-10-(4-methoxyphenyl)-2-methyl-2-(4-methyl-3-penten-1-yl)-8H-Pyrano[2,3-f]quinolin-8-one" | 2.0931 | - | - | - | - | - |
| Folinic acid | 1.9384 | C20H23N7O7 | 473.1658 | 0001562 | 149436 | C03479 |
| (+)-Tetrandrine | 1.8952 | C38H42N2O6 | NA | NA | 11844 | C09654 |
| Evorine | 1.8621 | - | - | - | - | - |
| Rutagravine | 1.7939 | C19H17NO5 | 339.1106 | 0033446 | 5465778 | NA |
| PS 24:5 | 1.7723 | - | - | - | - | - |
| "beta-D-Glucopyranoside, 2-methoxy-4-[(2R,3R,4S)-tetrahydro-4-hydroxy-4-[(4-hydroxy-3-methoxyphenyl)methyl]-3-(hydroxymethyl)-2-furanyl]phenyl" | 1.7397 | - | - | - | - | - |
| 2-Hydroxy-butanoic acid | 1.7373 | C4H8O3 | 104.0473 | 0000008 | 440864 | C05984 |
| Aluminum neocoprogen I | 1.7041 | - | - | - | - | - |
| Norfloxacin | 1.5833 | C16H18FN3O3 | 319.1332 | 0015192 | 4539 | C06687 |
| Naltrexone-3-glucuronide | 1.5664 | - | - | - | - | - |
| Quinapril | 1.539 | C25H30N2O5 | 438.2154 | 0015019 | 54892 | C07398 |
| Carbadox | 1.5183 | C11H10N4O4 | 262.0702 | 0031762 | 135511839 | NA |
| Rescinnamine | 1.5131 | C35H42N2O9 | 634.2890 | 0015311 | 5280954 | C06540 |
| "alpha-(acetyloxy)-4-(3-furanyl)-1,4,4a,5,6,6a,7,8,9,10,11,12-dodecahydro-11-hydroxy-4a,7,9,9-tetramethyl-10-[[(2E)-2-methyl-1-oxo-2-buten-1-yl]oxy]-2,13-dioxo-7,11-Methano-2H-cycloocta[3,4]benzo[1,2-c]pyran-8-acetic acid, methyl ester" | 1.4604 | - | - | - | - | - |
| D-Ribose | 1.4556 | C5H10O5 | 150.0528 | 0000283 | 5779 | C00121 |
| Sildenafil | 1.4394 | C22H30N6O4S | 474.2049 | 0005039 | 5212 | C07259 |
| Margrapine B | 1.4238 | C22H25NO7 | 415.1631 | 0031279 | 102003359 | NA |
| Itraconzaole | 1.4104 | - | - | - | - | - |
| cyclo-(Ala-4-hydroxy-Pro) | 1.4094 | - | - | - | - | - |
| Succinic acid | 1.3276 | C4H6O4 | 118.0266 | 0000254 | 1738118 | C00042 |
| Pirbuterol | 1.3010 | C12H20N2O3 | 240.1473 | 0015407 | 4845 | C07807 |
| Thr-Leu | 1.3009 | C10H20N2O4 | 232.1423 | 0259044 | NA | NA |
| Cyclo(glycyltryptophylprolylglycylvalylglycyltyrosyl) | 1.2816 | - | - | - | - | - |
| 4-Hydroxybenzaldehyde | 1.2611 | C7H6O2 | 122.0367 | 0011718 | 126 | C00633 |
| Naratriptan | 1.1055 | C17H25N3O2S | 335.1667 | 0015087 | 4440 | C07792 |
| "1,6-Dihydro-1-(3-methoxyphenethyl)-6-oxonicotinic acid" | 1.0904 | C15H15NO4 | NA | NA | 17396086 | C15089 |
| Lonchocarpenin | 1.0761 | C27H28O6 | NA | NA | 12674 | C10491 |
| (R)-Rutaretin 1-(6-sinapoylglucoside) | 1.0737 | C31H34O14 | 630.1948 | 0039041 | 14033991 | NA |
| Chlorhexidine | 1.0658 | C22H30Cl2N10 | 504.2031 | 0015016 | 2713 | C06902 |
| 5-Hydroxystreptomycin | 1.0652 | C21H39N7O13 | NA | NA | 96023916 | C17571 |
| PS 27:6 | 1.0642 | - | - | - | - | - |
| "(1S,3R,13S,14S,17S,18R,19R,20S,21S,23R,24R,25S)-20-(Acetoxymethyl)-18,25-dihydroxy-3,13,14,25-tetramethyl-6,15,22-trioxo-2,5,16-trioxa-11-azapentacyclo[15.7.1.0~1,20~.0~3,23~.0~7,12~]pentacosa-7,9,11-triene-19,21,24-triyl triacetate" | 1.0583 | - | - | - | - | - |
| Muricarpone B | 1.0509 | - | - | - | - | - |
| Hypoxanthine | 1.0505 | C5H4N4O | 136.0385 | 0000157 | 790 | C00262 |
| PS 19:3 | 1.0458 | - | - | - | - | - |
| PC 6:0 | 1.0454 | - | - | - | - | - |
| PS 18:3 | 1.0435 | - | - | - | - | - |
| "Methyl [(1S,3S,7R,8R,9R,12S,13S)-13-(3-furyl)-6,6,8,12-tetramethyl-17-methylene-5,15-dioxo-2,14-dioxatetracyclo[7.7.1.0~1,12~.0~3,8~]heptadec-7-yl]acetate" | 1.0398 | - | - | - | - | - |
| "3,7,3,4-Tetrahydroxyflavanone" | 1.0356 | - | - | - | - | - |
| Purothionin AII | 1.0318 | C26H36N2O9 | 520.2420 | 0039001 | 245869 | NA |
| iso-Roridine E | 1.0247 | - | - | - | - | - |
| Gravacridonetriol | 1.0157 | C19H19NO6 | 357.1212 | 0029330 | 21586648 | NA |
| Thesinine 4-O-glucoside | 1.0147 | C23H31NO8 | 449.2049 | 0039900 | 131752752 | NA |
| "(5S,8S,9R)-8-Benzoyl-2-[(1S,2S,3Z)-1,2-dihydroxy-3-hexen-1-yl]-8,9-dihydroxy-3-methyl-1-oxa-7-azaspiro[4.4]non-2-ene-4,6-dione" | 1.0078 | - | - | - | - | - |
| 4-Deoxyphysalolactone | 0.9995 | C28H39ClO7 | 522.2384 | 0034346 | 13743195 | NA |
| Kanokoside A | 0.9903 | C21H32O12 | 476.1893 | 0035635 | 5316533 | C17428 |
| 4-Hydroxyhomopterocarpin | 0.9867 | C17H16O5 | 300.0997 | 0033142 | 834 | C10461 |
| 2-Hydroxy-desipramine glucuronide | 0.9650 | C24H30N2O7 | 458.2053 | 0060716 | 71748973 | NA |
| "Phenyl 6-O-[(2R,3R,4R)-3,4-dihydroxy-4-(hydroxymethyl)tetrahydro-2-furanyl]-beta-D-glucopyranoside" | 0.9646 | - | - | - | - | - |
| Olanzapine | 0.9498 | C17H20N4S | 312.1408 | 0005012 | 135398745 | C07322 |
| 15-Acetyl-4-deoxynivalenol | 0.9466 | C17H22O7 | 338.1365 | 0033444 | 135402018 | NA |
| Loxapine | 0.9436 | C18H18ClN3O | 327.1138 | 0014552 | 3964 | C07104 |
| Gibberellin A1 | 0.9420 | C19H24O6 | NA | NA | 4116 | C00859 |
| 12(13)-EpOME-d | 0.9335 | - | - | - | - | - |
| Rhaponticin | 0.9317 | C21H24O9 | 420.1420 | 0304731 | NA | C10288 |
| Eupatoroxin | 0.9285 | C20H24O8 | NA | NA | 11634 | C09443 |
| D-Fructosazine | 0.9239 | C12H20N2O8 | 320.1219 | 0035284 | 11956719 | NA |
| FIPI | 0.9158 | - | - | - | - | - |
| Oxynarcotine | 0.9128 | C22H25NO8 | 431.1580 | 0030247 | 5320347 | NA |
| Gibberellin A76 | 0.9054 | C19H24O7 | 364.1522 | 0036898 | 14583171 | NA |
| L-Gulonic-gamma-lactone | 0.8967 | C6H10O6 | 178.0477 | 0003466 | 439373 | C01040 |
| 2-Deoxymugineic acid | 0.8933 | C12H20N2O7 | 304.1270 | 0033909 | 4641377 | C15485 |
| 4-O-Phosphohygromycin B | 0.8920 | C20H38N3O16P | NA | NA | 135626317 | C19850 |
| Acetamiprid | 0.8858 | C10H11ClN4 | 222.0672 | 0247906 | 213021 | C18507 |
| SN38 glucuronide | 0.8760 | C28H28N2O11 | 568.1693 | 0060511 | 443154 | C11376 |
| Hydramethylnon | 0.8738 | C25H24F6N4 | NA | NA | 13177 | C10994 |
| D-Pyrrolidine-2-carboxylic acid | 0.8634 | C5H9NO2 | 115.0633 | 0003411 | 8988 | C00763 |
| N-Acetyl-D-mannosamine | 0.8625 | C8H15NO6 | 221.0899 | 0001129 | 11096158 | C00645 |
| S-Succinyldihydrolipoamide | 0.8595 | C12H21NO4S2 | 307.0911 | 0001177 | 439425 | C01169 |
| "alpha-(1,2-Dihydroxyethyl)-1,2,3,4-tetrahydro-7-hydroxy-9-methoxy-3,4-dioxocyclopenta[c][1]benzopyran-6-acetaldehyde" | 0.8588 | C17H16O8 | 348.0845 | 0062442 | 53297447 | C19592 |
| 5-Hydroxyxanthotoxin | 0.8587 | C17H24O7 | 340.1522 | 0036157 | 10337297 | C02951 |
| D-Xylose | 0.8585 | C5H10O5 | 150.0528 | 0000098 | 135191 | C00181 |
| p-Coumaroyl quinic acid | 0.8566 | C16H18O8 | 338.1001 | 0029681 | 9945785 | C12208 |
| Creatinine | 0.8461 | C4H7N3O | 113.0589 | 0000562 | 588 | C00791 |
| N-cis-Caffeoyltyramine | 0.8314 | C17H17NO4 | 299.1157 | 0033026 | 9994897 | NA |
| Dl-Threonine | 0.8305 | C4H9NO3 | 119.0582 | 0000167 | 6288 | C00188 |
| Lactose | 0.8303 | C12H22O11 | 342.1162 | 0000186 | 84571 | C00243 |
| 19-Hydroxy-8-O-methyltetrangulol | 0.8303 | C20H14O5 | NA | NA | 582790 | C12400 |
| "3-Ethoxyandrosta-3,5-dien-17beta-ol propanoate" | 5.3716 | C24H36O3 | NA | NA | 17396054 | C15057 |
| Linagliptin (BI-1356) | 3.2366 | - | - | - | - | - |
| Gibberellic acid | 2.7822 | C19H22O6 | 346.1416 | 0003559 | 6466 | C01699 |
| Alfuzosin | 2.5919 | C19H27N5O4 | 389.2063 | 0014490 | 2092 | NA |
| p-Fluorophenylalanine | 0.9171 | C9H10FNO2 | 183.0695 | 0246423 | 4654 | C11217 |


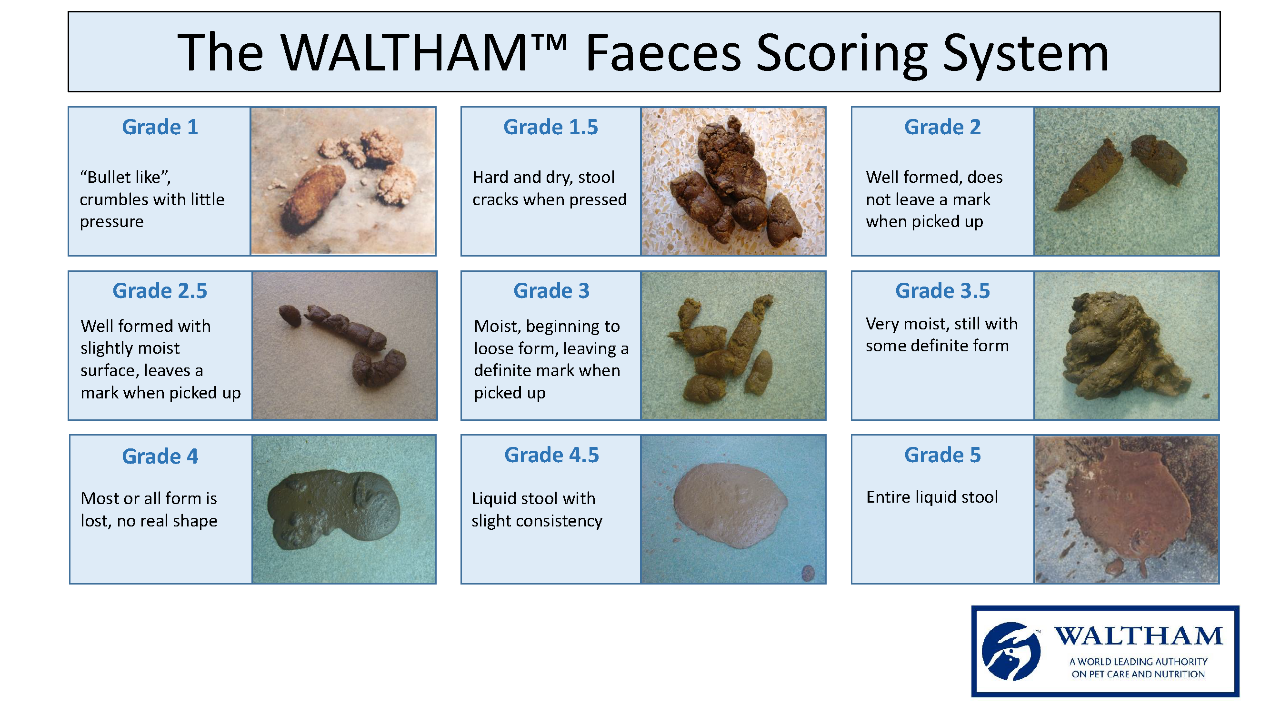
 **SFigure1.** The Waltham® faeces scoring system**.**


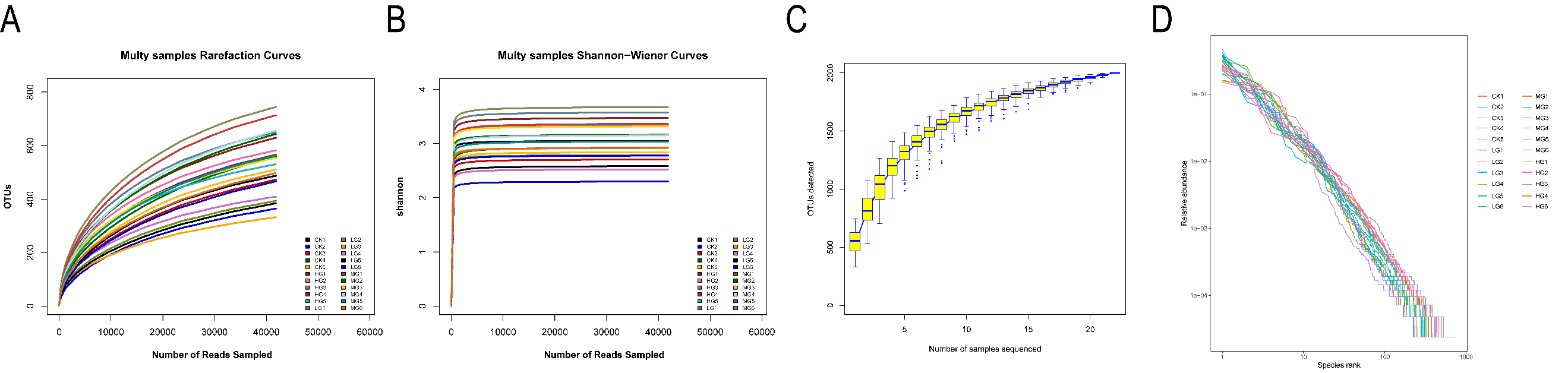
**SFigure2.** Alpha diversity analysis of beagles supplemented different concentrations of the probiotic GLP06.


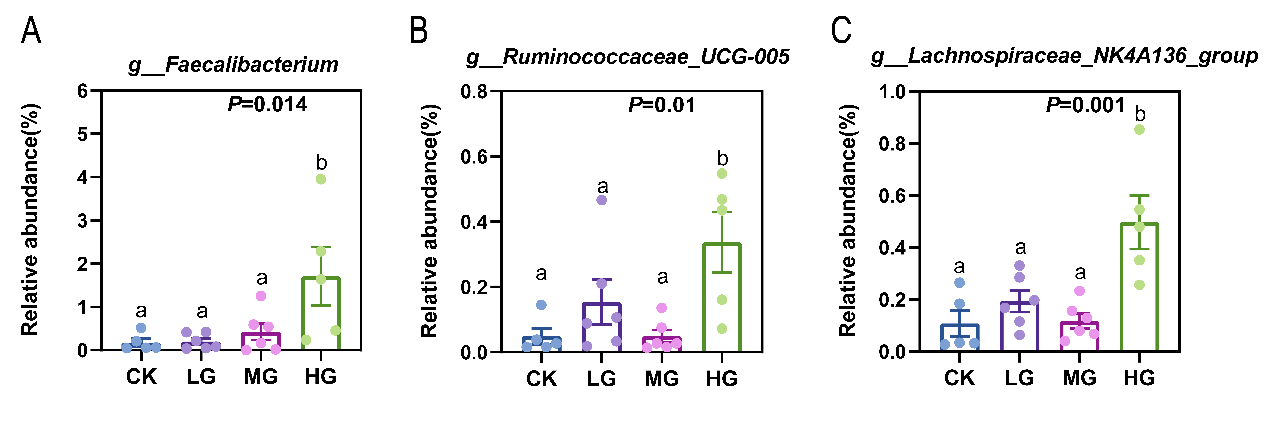
**SFigure3.** Differential species analysis of beagles supplemented different concentrations of the probiotic GLP06.
