# Supplementary figures and images for: Impact of Pediococcus acidilactici GLP06 supplementation on gut microbes and metabolites in adult beagles: a comparative analysis
Source: Front Microbiol. 2024 Apr 3;15:1369402. doi: 10.3389/fmicb.2024.1369402 (PMC11021720; doi:10.3389/fmicb.2024.1369402)

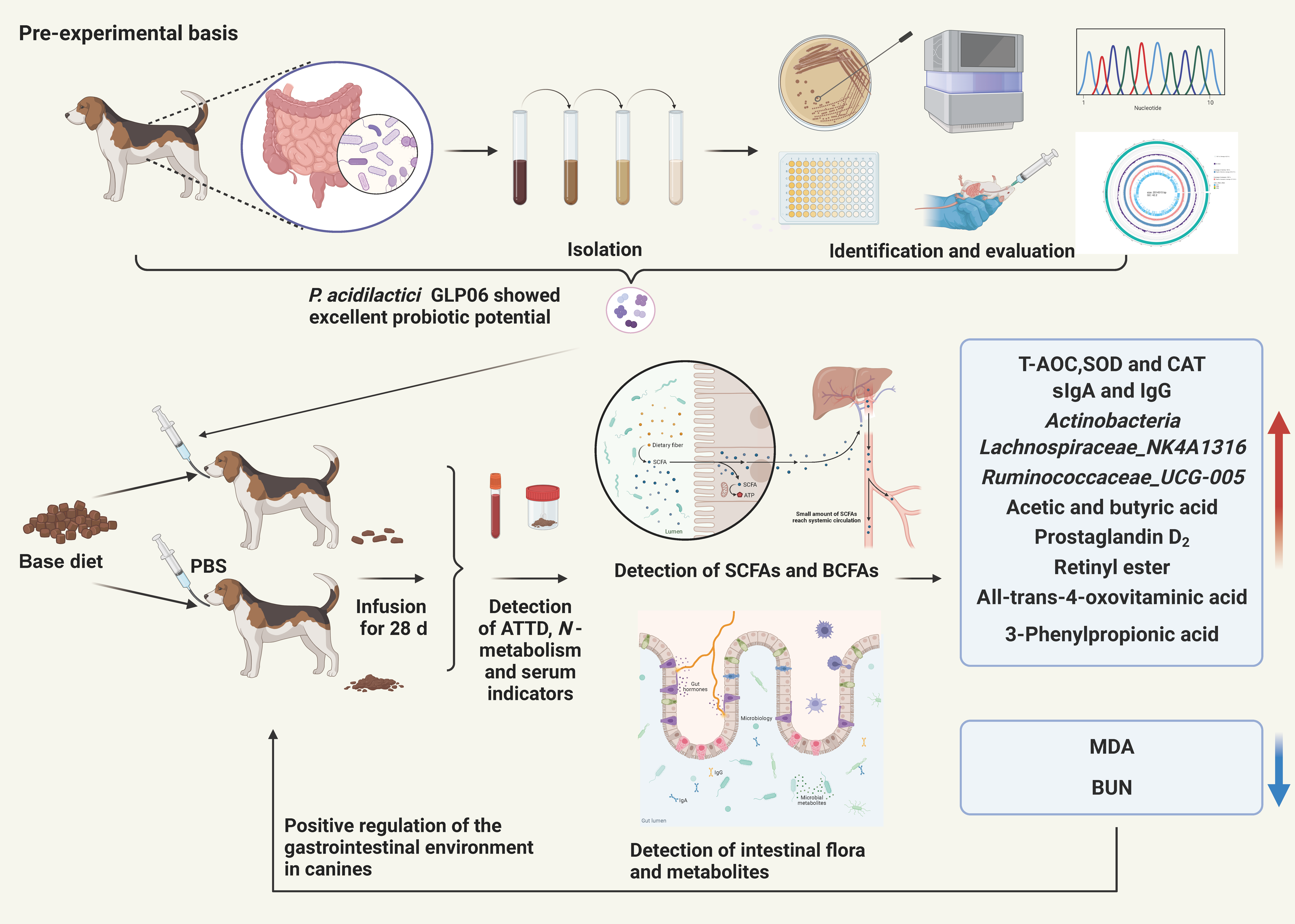

Supplement: Supplementary file 2 [file Image_1.TIFF]
